# DUOX-Generated Intestinal ROS Restrict Spore Adhesion and Invasion of *Nosema bombycis* in Silkworm *Bombyx mori*

**DOI:** 10.3390/biology15171544

**Published:** 2026-09-04

**Authors:** Hanjun Wang, Tingyue Huang, Pengfei Wang, Qianmin Hai, Rui Ma, Man Yu, Xiaoqun Dang, Jinshan Xu, Chunfeng Li, Guoqing Pan, Zhengang Ma, Zeyang Zhou

**Affiliations:** 1 Key Laboratory of Pollinator Resources Conservation and Utilization of the Upper Yangtze River, Ministry of Agriculture and Rural Affairs, Chongqing Normal University, Chongqing 401331, China; 2 Chongqing Key Laboratory of Vector Control and Utilization, College of Life Sciences, Chongqing Normal University, Chongqing 401331, China; 3 State Key Laboratory of Resource Insects, Southwest University, Chongqing 401331, China

**Keywords:** *Bombyx mori*, *Nosema bombycis*, intestinal immunity, DUOX-ROS pathway

## Abstract

The intestinal immune system is essential for insects to defend against pathogenic infections, while the dual oxidase-reactive oxygen species (DUOX-ROS) pathway is a key component. However, the role of the DUOX-ROS pathway in *Bombyx mori* against *Nosema bombycis*, a significant pathogen of this species, remains poorly characterized. In this study, four core components of the DUOX-ROS pathway in *B. mori* (*BmGαq*, *BmPLCβ1*, *BmPLCβ4*, and *BmDUOX*) were identified using bioinformatics. Pathogen induction experiments showed significant upregulation of *BmDUOX* and increased ROS production following *N. bombycis* infection. Immunofluorescence and antibody-blocking assays demonstrated that BmDUOX localizes to the plasma membrane and is essential for inhibiting spore adhesion and invasion. This study provides the first systematic evidence of DUOX-ROS-mediated anti-microsporidian immunity in *B. mori*, which may provide a theoretical basis for the future development of ROS-based disease control strategies in sericulture.

## Simple Summary

The silkworm, *Bombyx mori*, is the foundation of the global silk industry, but it faces serious threats, for example, the microsporidian parasite *Nosema bombycis*. This pathogen invades the host through the gut, making the intestinal immune system a key defense. In this study, the role of the DUOX-ROS immune pathway in the silkworm’s response to *N. bombycis* infection was investigated. We found that the infection significantly upregulated *BmDUOX* expression, leading to elevated ROS production in the silkworm gut. Moreover, modulation of this pathway was associated with altered spore adhesion and invasion, suggesting its involvement in restricting the initial infection of gut cells. Our findings reveal a natural defense mechanism that could inspire new strategies to protect silkworms from *N. bombycis*, potentially reducing economic losses in sericulture. Understanding this process also provides broader insights into how insect guts defend against pathogens.

## 1. Introduction

Insects inhabit a highly complex environment, and their gut epithelial cells are continually exposed to a variety of microbial communities [1,2]. While some microbes provide benefits to the host, others function as pathogens, leading to individual mortality and potentially causing population collapse during severe infections [3]. Throughout evolutionary history, insects have developed complex defense mechanisms to combat pathogenic invasion. Among these, the intestinal barrier acts as a crucial frontline defense that eliminates invading pathogens while maintaining symbiotic microbial homeostasis [4]. Studies indicate that insect gut immunity relies on a synergistic interaction between physical barriers and antibacterial effector molecules, primarily antimicrobial peptides (AMPs) and reactive oxygen species (ROS) [4,5,6].

The insect gut immune response relies on two main pathways: the production of AMPs by the IMD pathway and the production of microbicidal ROS by the DUOX-ROS pathway [7,8]. ROS, by-products of aerobic metabolism, mainly include hydrogen peroxide (H_2_O_2_), hydroxyl radicals (•OH), superoxide anions (O_2_•^−^), and other oxygen-derived molecules [9,10]. They play vital roles in developmental regulation, metabolic modulation, and maintaining the integrity of the reproductive system [9,11,12]. Due to its highly reactive nature, ROS have significant microbicidal effects in innate immunity. At appropriate concentrations, ROS act as a kind of powerful antimicrobial agents effectively eliminating invasive bacteria, fungi, and protozoan pathogens [13,14,15]. Notably, in the insect gut, the DUOX-ROS pathway discriminates pathogens from commensals by sensing microbe-derived uracil. Pathogenic microorganisms release uracil and activate DUOX to produce microbicidal ROS, whereas commensals do not produce uracil and thus do not trigger ROS responses, allowing them to colonize the gut stably [16,17]. However, the overgeneration of ROS has been demonstrated to harm important biomacromolecules, including DNA, proteins, and lipids [18], disrupting gut microbial balance and triggering apoptosis [17,19].

It has been demonstrated that ROS production is primarily regulated by dual oxidase (DUOX), a crucial enzyme activated during pathogenic challenges in insects, which plays a vital role in controlling opportunistic pathogens in the gut [13,20,21]. For example, in *Drosophila*, recent studies have shown that there are two DUOX-regulatory pathways that function in efficient pathogen-induced ROS production: the “DUOX-expression pathway” and the “DUOX-activity pathway” [20,21]. The “DUOX-expression pathway” regulates *DUOX* expression by sensing pathogen-derived peptidoglycan (PG) ligands through membrane-bound peptidoglycan recognition proteins (PGRPs), which sequentially activate the IMD-MEKK1-MKK3-p38-ATF2 cascade [21]. Furthermore, the Mesh, a gut-membrane-associated protein in *Drosophila*, can also regulate the expression of *DUOX* via an Arrestin-mediated MAPK JNK/ERK phosphorylation cascade [22]. The “DUOX-activity pathway” modulates DUOX enzymatic activity through the Gαq-PLCβ-Ca^2+^ mediated signaling, which triggers the release of Ca^2+^ from the endoplasmic reticulum, thereby activating the enzymatic activity of DUOX and promoting ROS production [20]. In addition, the DUOX-ROS immune defense system has been shown to maintain intestinal microbial homeostasis in insects and regulate the dysbiosis of intestinal flora caused by pathogen invasion. Researchers have discovered that silencing the *BdDUOX* gene in *Bactrocera dorsalis* led to an imbalance in the gut bacterial community, which subsequently triggers *BdDUOX* expression and enhanced ROS production to suppress the excessive proliferation of opportunistic pathogens, thereby restoring microbial balance [23]. Furthermore, a recent study indicated that serotonin (5-hydroxytryptamine, 5-HT) in the guts of *Bactrocera dorsalis* and *Aedes aegypti* can influence the homeostasis of the gut microbiota by regulating the expression of *DUOX* [24]. These findings collectively highlight the crucial role of the DUOX-ROS pathway in pathogen defense and microbiota regulation, elucidating its significance in preserving insect gut health.

The DUOX-ROS pathway is well-characterized in model insects like *Drosophila*; however, its physiological roles and molecular mechanisms underlying the immune response in economically significant species, such as the silkworm, are still poorly understood. The silkworm, *Bombyx mori* (*B. mori*), a cornerstone species of the global silk industry with considerable economic value, is persistently threatened by pebrine disease. *Nosema bombycis* (*N. bombycis*), the first identified microsporidia, is the pathogen of pebrine disease in *B. mori*, which is responsible for the mortality in *B. mori* populations and considerable economic losses [25,26,27].

Current understanding of the molecular mechanisms by which *N. bombycis* invades its host remains incomplete, although at least two infection strategies have been documented [28]. The most well-characterized pathway involves direct injection via polar tube extrusion. Upon receiving environmental stimuli, the mature spore rapidly discharges its polar tube, which penetrates the host cell plasma membrane and serves as a conduit for the injection of the infective sporoplasm directly into the host cell cytoplasm. An alternative entry route has also been revealed, in which microsporidia exploit host phagocytic or endocytic uptake to gain access to the intracellular environment. In this pathway, spores are internalized by the host cell via phagocytosis or endocytosis, followed by intracellular germination and polar tube extrusion within the phagosome; the parasite subsequently escapes from the maturing phagosome or lysosome into the host cytoplasm to establish infection. The specialized polar tube apparatus, responsible for the direct injection pathway, endows microsporidia with a powerful “injection-based” mode of infection, conferring a remarkable advantage in infection efficiency. In nature, *N. bombycis* is transmitted *per os* via contaminated food, primarily targeting the midgut epithelial cells of *B. mori* [29]. Thus, similar to other insects, the intestinal epithelial cells of *B. mori* likely serve as a critical defense barrier against microsporidian invasion. While considerable attention has been given to the infection mechanisms of *N. bombycis* [30,31,32,33,34,35], host innate immune responses, particularly the DUOX-ROS pathway, have received far less study. Previous studies have identified BmDUOX, a key enzyme of the DUOX-ROS pathway in *B. mori* [36]. The expression level of *BmDUOX* in the midgut was significantly upregulated upon *per os* challenge with *Escherichia coli* or *Bombyx mori* nucleopolyhedrovirus (BmNPV), suggesting that the DUOX-ROS pathway may participate in intestinal immune defense against multiple classes of pathogens in the silkworm. Nevertheless, whether this pathway responds to microsporidian infection, and the precise mechanisms by which it might operate in this context, remain to be fully elucidated.

In this study, we identified core components of the DUOX-ROS pathway in *B. mori*—*BmGαq*, *BmPLCβ1*, *BmPLCβ4*, and *BmDUOX*—using bioinformatic tools. Immunofluorescence and antibody-blocking assays confirmed that BmDUOX localizes to the plasma membrane of *B. mori* midgut and is essential for inhibiting *N. bombycis* spore adhesion and invasion. Pathogen induction experiments revealed significant upregulation of *BmDUOX* in the midgut following *N. bombycis* infection, indicating a pathogen-specific activation of this pathway. Cell induction experiments demonstrated that both *N. bombycis* spores and the soluble microbial extract (NbSME) robustly induce ROS production in BmE cells. Based on these findings, we propose a putative intestinal immune model in which *B. mori* may recognize soluble molecular components from *N. bombycis,* which could trigger the Gαq-PLCβ-Ca^2+^-dependent DUOX-ROS signaling pathway to produce ROS. These ROS may impair the functional integrity of spore surface proteins, thereby limiting spore adhesion and invasion to host cells. This study offers a new perspective for elucidating the intestinal immune mechanism of *B. mori* against microsporidian infection.

## 2. Materials and Methods

### 2.1. Bioinformatics Analysis of Genes Associated with DUOX-ROS Signaling Pathway in B. mori

Here, genomic retrieval and sequence characterization analyses of Gαq, PLCβ, and DUOX proteins involved in the DUOX-ROS signaling pathway in *B. mori* were conducted. Using previously reported sequences of *Drosophila melanogaster* Gαq (GenBank accession: AAA28460.1), PLCβ (GenBank accession: AAA28724.1), and DUOX (GenBank accession: NP_608715.2) as query sequences, BLASTP searches were performed against the *B. mori* genome database (SilkDB) with a threshold of E-value < 10^−6^. The candidate sequences obtained from these searches were further validated through reciprocal BLASTP alignment against the NCBI GenBank database.

To comprehensively analyze the sequence features of BmGαq, BmPLCβ1, BmPLCβ4 and BmDUOX, we employed multiple bioinformatics tools. Chromosome viewer (https://silkdb.bioinfotoolkits.net/main/chromosome/ (accessed on 3 June 2026)) was used for genetic analysis of the chromosome distribution, and BmMDB (http://www.silkdb.org/microarray/ (accessed on 3 June 2026)) for microarray expression data analysis. Molecular weight and isoelectric point (pI) were predicted using ExPASy (https://web.expasy.org/compute_pi/ (accessed on 6 June 2026)). ProtParam (http://web.expasy.org/protparam/ (accessed on 6 June 2026)) was utilized to analyze protein charge distribution and hydrophobicity. TMHMM 2.0 (http://www.cbs.dtu.dk/services/TMHMM-2.0/ (accessed on 6 June 2026)) was used to predict transmembrane regions, while SignalP 6.0 (https://services.healthtech.dtu.dk/services/SignalP-6.0/ (accessed on 6 June 2026)) was used to identify potential signal peptide sequences. WegoLoc (weighted gene ontology term-based subcellular localization prediction, http://www.btool.org/wegoloc (accessed on 8 June 2026)), DeepLoc-2.1 (https://services.healthtech.dtu.dk/services/DeepLoc-2.1/ (accessed on 8 June 2026)) and Euk-mPLoc 2.0 (Predicting subcellular localization of eukaryotic protein, http://www.csbio.sjtu.edu.cn/bioinf/euk-multi-2/ (accessed on 8 June 2026)) were employed to predict the subcellular localization of proteins. The analysis of gene tissue expression profiles and temporal expression profiles was completed in SilkDB 3.0 (https://silkdb.bioinfotoolkits.net/main/species-info/-1 (accessed on 3 June 2026)).

### 2.2. Cloning, Expression, and Preparation of Anti-BmDUOX_OM and Anti-BmDUOX_POD Serums

Using the TMHMM Server, HMMTOP, and TMpred, the transmembrane regions of BmDUOX were predicted. The results indicated that the amino acid residues 49–176 at the N-terminus of the BmDUOX protein were predicted to be part of the extracellular region, referred to as BmDUOX_OM. The full-length peroxidase domain of the BmDUOX protein was then predicted by the Pfam and NCBI database BlastP tool. Combining the results from both predictions, the amino acids 1–518 at the N-terminus of the BmDUOX protein were selected for further study, referred to as BmDUOX_POD.

Subsequently, primers were designed by Premier 5.0, which are specific to the *BmDUOX_OM* (*BmDUOX_OM*-F: 5′-ATATGGATCCGAGTCCGGATGTCCTATCGA-3′; *BmDUOX_OM*-R: 5′-GCGCGTCGACTCATTAAGCGTAATCTGGAACATCGTATGGGTACATTGGATTTTGATTGGTACGTG-3′) and the *BmDUOX_POD* (*BmDUOX_POD*-F: 5′-ATATGGATCCATGGCCGGTCCTGAGAGACC-3′; *BmDUOX_POD*-R: 5′-GCGCGTCGACTCATTAGTGATGATGATGATGATGGTTTCCCTCAAAATAATCGT-3′) gene segments. For *BmDUOX_POD* primers, *Bam*H I restriction site was added in the forward primer, and the His-tag coding sequence together with *Sal* I restriction site were introduced in the reverse primer. For *BmDUOX_OM* primers, *Bam*H I and *Sal* I restriction sites were added to forward and reverse primers, respectively, and the HA-tag sequence was incorporated into the reverse primer. All recombinant plasmids constructed using these primers were validated by DNA sequencing.

Total RNA was extracted using TRIzol reagent (Sangon, Shanghai, China) and then reverse transcribed into cDNA with a reverse transcription kit (TaKaRa, Shanghai, China). The target genes were amplified using the cDNA as a template along with the specific primers mentioned above. The PCR conditions were as follows: 94 °C for 5 min; 94 °C for 40 s, 60 °C for 1 min, 72 °C for 90 s, 35 cycles; 72 °C for 10 min, and the period was held at 12 °C. Each PCR mixture contained 5.0 μL of 10 × HiFi Taq buffer, 4.0 μL of 10 mmol/L dNTP, 0.2 μL of 2.5 U/μL HiFi Taq, 0.80 μL of each 10 μmol/L primer and 0.5 μL of cDNA, followed by the addition of ddH_2_O to bring the final volume to 50 μL. The amplified products were separated using a 1.0% agarose gel, after which the fragments were ligated into the pColdI vector and transformed into *E. coli* strain *Trans* 5α chemically competent cells. Once the DNA construct was verified by sequencing (Invitrogen, Shanghai, China), the recombinant expression vector was transformed into *E. coli Rosetta* (DE3) cells for prokaryotic expression. The obtained recombinant proteins were then induced at 16 °C for 24 h in the presence of 0.1 mmol/L IPTG and confirmed by Western blotting. Subsequently, the target recombinant proteins were purified using Ni-NTA affinity chromatography (Qiagen, Beijing, China). SDS-PAGE was subsequently employed to assess the purity of the recombinant proteins.

The concentrations of the purified recombinant proteins rBmDUOX_POD and rBmDUOX_OM were determined using a Bradford protein assay kit (Beyotime, Beijing, China). Based on the measured concentrations, the injection volume was calculated to deliver a dose of 100 μg per mouse. Ten mice were evenly divided into two groups and immunized with rBmDUOX_POD or rBmDUOX_OM mixed with Freund’s adjuvant (1:1; Sigma-Aldrich, St. Louis, MO, USA), respectively. Five additional mice were injected with phosphate-buffered solution (PBS) as a negative control. The mice were immunized four times in total (complete Freund’s adjuvant was used for the first immunization, and incomplete Freund’s adjuvant was used for the subsequent ones) at 7-day intervals. Seven days after the final immunization, blood was collected from each mouse under isoflurane anesthesia. The blood samples were allowed to clot at 37 °C for 1 h and then kept at 4 °C overnight. After centrifugation, the upper layer containing the serum was collected and stored at −80 °C. Western blotting was then employed to analyze the specificity of anti-BmDUOX_OM serum and anti-BmDUOX_POD serum.

### 2.3. Analysis of the Subcellular Localization of the BmDUOX Protein

#### 2.3.1. Immunohistochemical Analysis

Paraffin sections of midgut tissue from normally feeding *B. mori* were prepared following He Yuanli’s procedure [37]. The slices were boiled in 0.01 mol/L citric acid buffer (pH 6.0) for 15–20 min, then cooled to room temperature and washed three times with PBS. The sections were subsequently incubated with sheep serum (10% *v*/*v*) at 37 °C for 1 h. After washing with PBS three times, the sample was treated with anti-BmDUOX_OM serum (1:200) and FITC-conjugated goat anti-mouse IgG (1:64) at 37 °C for 1 h and 1.5 h, respectively. Two negative-control groups were included under identical experimental conditions: one using 1:200 (*v*/*v*) diluted mouse negative serum, and the other using 1:200 (*v*/*v*) diluted mouse pre-immune serum. Following three washes with PBS, DNA was stained with DAPI for 20 min. An antifade mounting medium was then added dropwise after washing three times with PBS, and slices were examined using an Olympus FV1200 laser confocal microscope (OLYMPUS, Tokyo, Japan) after preparation.

#### 2.3.2. Immunofluorescence Analysis (IFA)

BmE cells cultured under normal conditions were passaged onto cell slides. After incubating overnight at 28 °C, the cells were treated with 4% (*v*/*v*) Triton X-100 for 10 min. Following three washes with PBS, the cells were blocked with 50 μL of blocking solution (containing 5% (*v*/*v*) BSA and 10% (*w*/*v*) goat serum in PBST) for 1 h at room temperature. The cells were then cleaned with PBS three times for 5 min each. Next, the BmE cells were incubated with mouse anti-BmDUOX_OM serum (1:400) as the primary antibody for 1 h at room temperature. The negative control group was treated with 1:400 (*v*/*v*) diluted mouse negative serum. After three washes with PBS, all cells were treated with FITC-conjugated goat anti-mouse IgG (1:64) as the secondary antibody for 1 h at room temperature. Following three washes with PBS, the DNA was stained with DAPI (1:1000) and kept in the dark for 20 min. An antifade mounting medium was then added dropwise after five washes with PBS, with each wash lasting 5 min. The prepared slides were then examined using an OLYMPUS BX-52 upright fluorescence microscope (OLYMPUS, Tokyo, Japan) after preparation.

#### 2.3.3. Membrane Protein Extraction

The extraction of membrane proteins from the midgut of silkworm larvae and BmE cells was conducted according to the operating steps of the Membrane and Cytosol Protein Extraction Kit (Beyotime, Beijing, China). In this kit, Reagent A is a mild detergent-based lysis buffer supplemented with protease and phosphatase inhibitors; Reagent B is a solubilization buffer containing a potent detergent for membrane protein extraction. All procedures were performed on ice at 4 °C to prevent protein degradation.

For midgut tissue samples, midguts from ten *B. mori* larvae (3rd day of 5th instar) were pooled to obtain approximately 100 mg of tissue as one biological sample. The tissues were minced with scissors on ice, resuspended in 1 mL of Reagent A (supplemented with PMSF), and incubated on ice for 10–15 min. The suspension was then homogenized in a pre-cooled glass homogenizer with 30–50 strokes. To monitor homogenization efficiency, 2–3 µL of the homogenate was placed on a glass slide and examined under a microscope to ensure that 70–80% of the cells had lost their intact morphology and perinuclear rings. The homogenate was centrifuged at 700× *g* for 10 min at 4 °C to remove nuclei and unbroken cells. The supernatant was carefully collected, leaving approximately 30–50 µL of residual liquid to avoid contamination, and then centrifuged at 14,000× *g* for 30 min at 4 °C. The cytoplasmic supernatant was discarded, and the membrane pellet was resuspended in 200–300 µL of Reagent B. The suspension was vigorously vortexed, incubated on ice for 5–10 min, and this vortex–incubation cycle was repeated 1–2 times to ensure complete solubilization. Finally, the sample was centrifuged at 14,000× *g* for 5 min at 4 °C, and the supernatant containing purified membrane proteins was collected and stored at −80 °C.

For BmE cells, 2 × 10^7^–5 × 10^7^ adherent cells were harvested, rinsed once with pre-cooled PBS, and detached using a trypsin-free EDTA-containing dissociation solution, followed by pipetting to detach the cells. The cell suspension was centrifuged at 600× *g* for 5 min at 4 °C, resuspended in pre-cooled PBS, counted using a hemocytometer, and re-centrifuged to completely remove residual supernatant. The cell pellet was resuspended in 1 mL of Reagent A (supplemented with PMSF) and incubated on ice for 10–15 min. The suspension was homogenized in a pre-cooled glass homogenizer with 30–50 strokes, and homogenization efficiency was confirmed using the same microscopic validation method and criteria described above. The homogenate was centrifuged at 700× *g* for 10 min at 4 °C to remove nuclei and unbroken cells, and the supernatant was collected with 30–50 μL of residual liquid left to avoid contamination. After centrifugation at 14,000× *g* for 30 min at 4 °C, the cytoplasmic supernatant was discarded. The membrane pellet was resuspended in 200–300 μL of Reagent B, vigorously vortexed, incubated on ice for 5–10 min, with the vortex–incubation cycle repeated 1–2 times. The mixture was centrifuged at 14,000× *g* for 5 min at 4 °C, and the membrane protein solution was collected and stored at −80 °C.

### 2.4. Pathogen Induction Experiment

#### 2.4.1. *B. mori* Midgut Challenged by *N. bombycis* Infection

PCR and sqRT-PCR were employed to analyze the transcriptional characteristics of *BmDUOX* in *N. bombycis* infected *B. mori*. The silkworms were raised on mulberry leaves in an artificial climate chamber at 25 °C and 75% humidity until the 3rd day of the 5th instar. Feeding was halted 12 h before the experiment began. The spores of *N. bombycis* were suspended in ddH_2_O to prepare the spore suspension. Spore viability was assessed by an in-vitro germination assay using alkaline-trigger medium; only spore preparations with germination efficiency over 70% were used for infections. Only morphologically intact, phase-bright spores were counted using a hemocytometer. Spores from the same batch were used for all biological replicates to eliminate batch-dependent infectivity variation. A 5 mL aliquot of *N. bombycis* spore suspension was then evenly applied to the back of mulberry leaves, which were subsequently air-dried naturally before being fed to the *B. mori*. Each larva in the experimental group was infected with 5 × 10^7^ spores of *N. bombycis,* as determined by hemocytometer counting. The larvae in the control group were treated with an equal amount of ddH_2_O instead of the suspension of spores. Then, midgut samples from the experimental and control groups were collected at 6, 12, 24 and 48 h post-infection. At each time point, 3 larvae per group were independently dissected and processed as individual biological replicates (*n* = 3 per time point per group), using dissecting scissors and forceps in ice-cold DEPC-treated water (Beyotime, Beijing, China). The dissected midguts were rinsed in DEPC-treated water to remove luminal contents, immediately frozen in liquid nitrogen, and stored at −80 °C for subsequent RNA extraction and cDNA synthesis. Sample size (*n* = 3) was determined based on preliminary experiments and is consistent with standard practices in silkworm midgut transcriptomic studies. The RT-PCR was then conducted using primers designed with Premier 5.0, which are specific to the *BmDUOX* and *Bmactin3* (*BmDUOX*_RT_F: 5′-CGTACCAATCAAAATCCAGCAA-3′; *BmDUOX*_RT_R: 5′-CATTATATTCAAAGCGCCAAGG-3′; *Bmactin3*_F: 5′-AACACCCCGTCCTGCTCACTG-3′; *Bmactin3*_R: 5′-GGGCGAGACGTGTGATTTCCT-3′), and carried out in a volume of 25 μL. Each PCR mixture consisted of 2.0 μL of 25 mmol/L MgCl_2_, 2.5 μL of 10 × Taq buffer, 2.0 μL of 10 mmol/L dNTP, 0.2 μL of 2.5 U/μL rTaq, 0.25 μL of 10 μmol/L each primer and 1.0 μL of cDNA extracted earlier, followed by the addition of ddH_2_O to bring the final volume to 25 μL. The PCR conditions were as follows: 94 °C for 5 min; 94 °C for 40 s, 55 °C for 40 s, 72 °C for 90 s, 25 cycles; 72 °C for 10 min, and the period ends at 12 °C.

Subsequently, sscDNA served as a template, and multiple PCR amplifications were conducted using the corresponding gene primers. The template amount was adjusted until the expression levels of the reference gene were consistent across all sampling times. This adjusted template amount was then used for subsequent PCR amplification. After PCR amplification, 10 μL of the product was detected by 1.0% agarose gel electrophoresis. The ImageJ software version 2.14.0 was utilized to measure the optical density values of the DNA strips, comparing the optical density values of *BmDUOX* with that of *Bmactin3* to ascertain the relative expression levels of *BmDUOX*.

#### 2.4.2. *N. bombycis* Spores Induce BmE Cells

BmE cells were routinely maintained in Grace’s insect medium (Invitrogen, San Diego, CA, USA) supplemented with 10% fetal bovine serum (Corning, New York, NY, USA) at 27 °C under normal atmospheric conditions. Normally cultured BmE cells were seeded into a 24-well plate until they grew to approximately 90% confluence. The experiment was divided into three groups: the negative control group, the *N. bombycis* infection group, and the positive control group, with three independent cell culture wells per group, each serving as an independent biological replicate (*n* = 3). Cells in the *N. bombycis* infection group were infected with mature *N. bombycis* spores at a multiplicity of infection (MOI) of 10:1 and incubated at 28 °C for 24 h. For the positive control group, ROSup reagent (50 mg/mL stock, diluted 1:1000 in PBS to a final concentration of 50 µg/mL) from the Reactive Oxygen Species Assay Kit (Beyotime, Shanghai, China) was added to each well (200 µL per well in a 24-well plate format). An equal volume of ddH_2_O was added to both the *N. bombycis* infection group and the negative control group. All three groups were then incubated at 28 °C for 30 min. Subsequently, ROS levels of cells from all three groups were measured simultaneously. ROS detection was performed strictly according to the Reactive Oxygen Species Assay Kit (Beyotime, Shanghai, China) instructions. For short-term stimulation (≤2 h), cells were loaded with DCFH-DA before stimulation; for long-term stimulation (≥6 h), cells were stimulated first followed by probe loading. DCFH-DA reagent from the Reactive Oxygen Species Assay Kit (Beyotime, Shanghai, China) was diluted 1:1000 in serum-free culture medium to a final concentration of 10 μmol/L for probe labeling. After removing the cell culture medium, 300 μL of the diluted DCFH-DA was added to each well, followed by incubation at 28 °C for 30 min. Cells were washed three times with sterile PBS to thoroughly remove any DCFH-DA that had not entered the cells. Then cells were harvested and analyzed via a microplate reader with excitation at 488 nm and an emission wavelength of 525 nm. Background fluorescence was subtracted using the negative control group in each experiment, and ROS levels were normalized to cell counts and expressed as relative DCF fluorescence intensity. ROSup reagent served as positive control for probe validation in each biological replicate. For fluorescence microscopy observation, cell nuclei were counter-stained with DAPI to verify comparable cell density among groups. Three independent biological replicates were performed for all ROS assays.

Meanwhile, approximately 1 × 10^4^ cells from each group were collected for DAPI staining. DAPI (1 mg/mL) was initially diluted 1:1000 with ddH_2_O, followed by washing the cells three times with PBS. The diluted DAPI was then added at a volume of 200 μL per well (for 24-well plate format), and the cells were stained in the dark at room temperature for 20 min. Subsequently, the cells were washed five times with PBS, with each wash lasting 5 min. After preparing the slides, an appropriate amount of anti-fade reagent was added, and the slides were promptly observed and photographed under a fluorescence microscope (OLYMPUS, Tokyo, Japan).

#### 2.4.3. Soluble Microbial Extract of *N. bombycis* (NbSME) Induce BmE Cells

Purified spores of *N. bombycis* (4 × 10^9^, as determined by hemocytometer) were collected, centrifuged at 3000 rpm for 5 min to remove the supernatant, and the spores were washed with ddH_2_O 2 to 3 times, after which the precipitation was collected. 400 μL of PBS-suspended spores were added, followed by 0.4 g of glass beads and PMSF to achieve a final concentration of 1.0 μmol/L. Soluble Microbial Extract of *N. bombycis* (NbSME) was obtained by vigorous oscillation on a vortex mixer for 3 to 9 h and centrifugation at 12,000 rpm for 10 min. The protein concentration was determined and subsequently analyzed by SDS-PAGE for future use. The experiment consisted of four groups: the control group, the NbSME induction group, the *Pichia pastoris* (GS115) SME induction group, and the positive treatment group, with three independent cell culture wells per group, each serving as an independent biological replicate (*n* = 3). First, the BmE cells were cultured in 24-well plates. Then, a final concentration of 10 μg/mL NbSME was added to each well of the NbSME induction group. Simultaneously, the GS115SME induction group received a final concentration of 10 μg/mL GS115SME in each well. The positive control group was supplemented with ROSUP (50 mg/mL stock, diluted 1:1000 in PBS to a final concentration of 50 μg/mL) at 200 μL per well in the 24-well plate format, while the negative control group consisted of the BmE cells cultured under normal feeding conditions. According to the above methods, ROS levels were detected in each group of cells.

To minimize potential confounding variables, all experimental groups within each assay were processed in parallel using identical culture conditions, reagent batches, and incubation protocols. Spore inoculations were performed with freshly prepared suspensions from the same purified spore stock, and all samples were collected and processed by the same operators following standardized procedures.

### 2.5. Antibody Blocking Assay

Normally cultured BmE cells were seeded into a 24-well plate until they grew to approximately 90% confluence. The ROS detection experiment consisted of five groups: the negative control group, the *N. bombycis* infection group, the positive control group, the OM_block group and the POD_block group. The BmE cells of the negative control group, the *N. bombycis* infection group and the positive control group were treated with 1:200 (*v*/*v*) diluted mouse negative serum, while the OM_block group and the POD_block group were treated with 1:200 (*v*/*v*) diluted anti-BmDUOX_OM serum or anti-BmDUOX_POD serum, respectively, and then incubated in a constant-temperature water bath at 28 °C for 2 h. After the antibodies had effectively blocked BmDUOX in the BmE cells, ROS levels were detected in each group of cells according to the above methods.

Next, BmE cells (~ 10^5^ cells per well) were seeded into 12-well cell culture plates. Cells were separated into three groups, with three independent culture wells per group serving as biological replicates (*n* = 3). The control group cells were treated with 1:200 (*v*/*v*) diluted mouse negative serum, while the experimental group cells were treated with 1:200 (*v*/*v*) diluted anti-BmDUOX_OM serum or anti-BmDUOX_POD serum as described above. Subsequently, 1 × 10^5^ *N. bombycis* spores were added to each cell culture well. Unattached spores were washed off with PBS after 2 h. At least 10 microscopic fields were examined, with each field containing no fewer than 3 cells. Then we quantified the average number of spores bound to host cell surfaces to assess spore adhesion levels. At 2 days post-infection, cell nuclei were stained with DAPI, and at least 10 fields were observed, ensuring that each field contained at least 3 cells. The average number of intracellular spores was quantified to indirectly evaluate the infection of BmE cells by *N. bombycis*.

### 2.6. Statistical Analysis

All data are presented as mean ± standard error of the mean (SEM) from at least three independent biological replicates, as specified in each experimental section. Statistical analyses were performed using GraphPad Prism 5.0 (GraphPad Software, San Diego, CA, USA). Two-group comparisons were carried out using Student’s *t*-test or Welch’s *t*-test. Multiple-group comparisons were conducted by one-way ANOVA with Tukey’s HSD post-hoc test, or Welch’s ANOVA coupled with Games-Howell post-hoc test when variances were unequal. Differences with *p* < 0.05 were considered statistically significant. Asterisks in figures denote significant differences for two-group comparisons, and different lowercase letters above bars indicate significant differences among multiple groups (*p* < 0.05).

## 3. Results

### 3.1. Characterization of the DUOX-ROS Pathway Genes BmGαq, BmPLCβ1, BmPLCβ4 and BmDUOX

**Figure 1:**
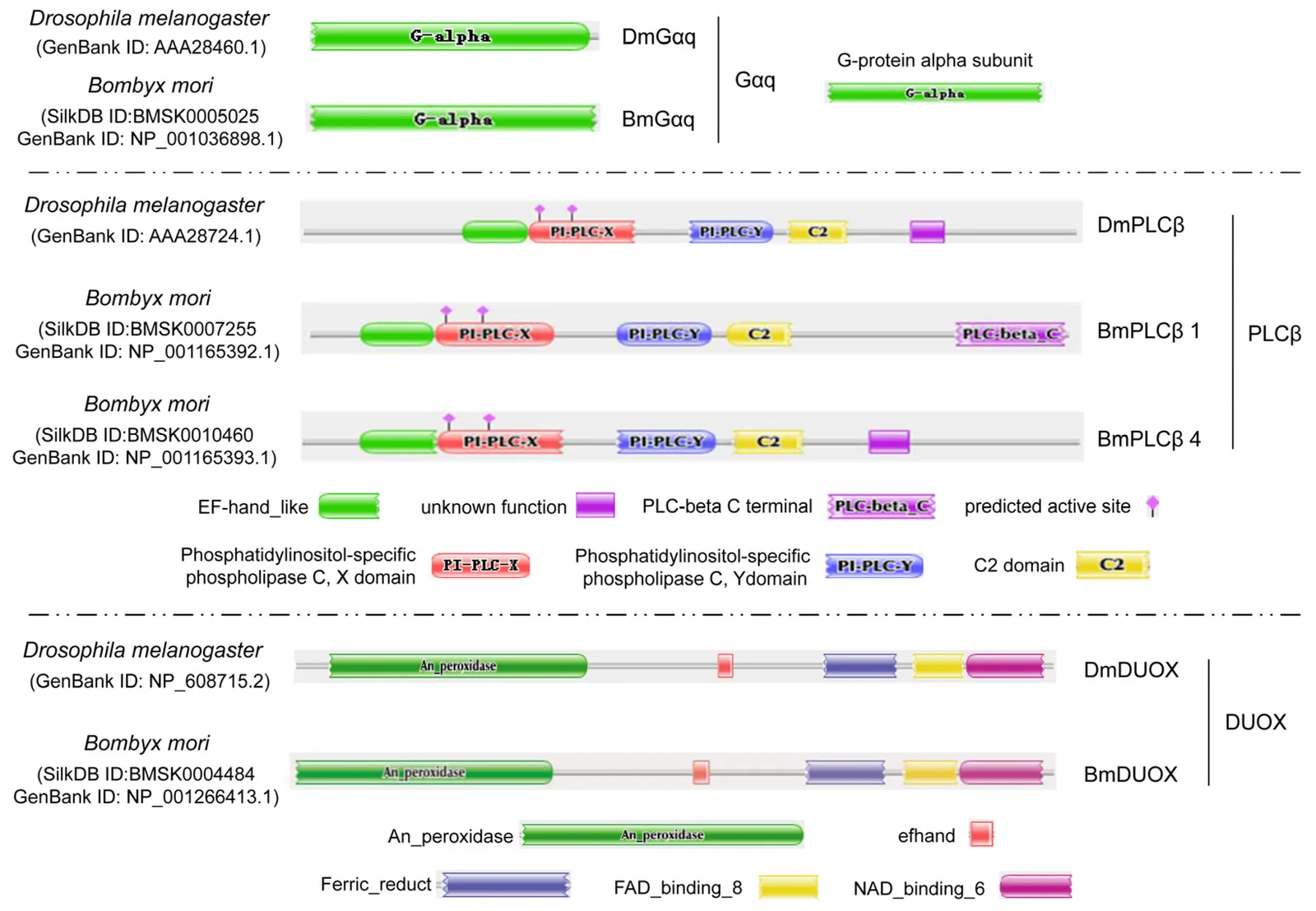
Domains of proteins involved in DUOX-ROS pathway in *B. mori*.

According to the aforementioned method, four gene sequences related to the DUOX-ROS pathway were obtained from the *B. mori* genome, including a *BmGαq* sequence (SilkDB3.0 accession: BMSK0005025). Two *BmPLCβ* sequences from the *B. mori* (SilkDB3.0 accession: BMSK0007255 and BMSK0010460) and one *BmDUOX* sequence (SilkDB3.0 accession: BMSK0004484) were also identified. To determine the results of the sequence alignment, the obtained sequences were analyzed using BlastP in the NCBI GenBank database, where homologous sequences were retrieved and the sequence information was verified. Ultimately, four genes related to the DUOX-ROS pathway were identified: *BmGαq*, *BmPLCβ1*, *BmPLCβ4* and *BmDUOX*. The conserved domain of the obtained sequences was analyzed using the Pfam database (Figure 1). As shown in Figure 1, the conserved domain of the DUOX-ROS-related proteins identified in this study aligns with the seed sequence and contains conserved functional structural regions, suggesting that they may perform similar functions.

Subsequently, online software and databases were utilized to analyze the sequence characteristics of *BmGαq*, *BmPLCβ1*, *BmPLCβ4* and *BmDUOX*, including chromosome distribution, genome location, exon number, signal peptide and transmembrane domain, molecular weight, and isoelectric point (Table 1). Results indicated that *BmGαq* encoded a 353-amino-acid protein with a calculated molecular mass of 41.49 kDa and a predicted pI of 5.15; *BmPLCβ1* encoded a 952-amino-acid protein with a calculated molecular mass of 107.86 kDa and a predicted pI of 6.72; *BmPLCβ4* encoded a 919-amino-acid protein with a calculated molecular mass of 106.10 kDa and a predicted pI of 6.76; *BmDUOX* encoded a 1428-amino-acid protein with a calculated molecular mass of 164.14 kDa and a predicted pI of 8.88. Additionally, the prediction results indicated that BmGαq, BmPLCβ1, and BmPLCβ4 all lacked signal peptides and transmembrane domains. However, BmDUOX possesses six transmembrane domains, which may suggest that the protein may perform functions in the cell membrane.

### 3.2. Prediction of Subcellular Localization of the Gene Encoding BmGαq, BmPLCβ1, BmPLCβ4 and BmDUOX

To gain a deeper understanding of the potential modes of action and positions of the proteins associated with the DUOX-ROS pathway in *B. mori*, WegoLoc (weighted gene ontology term-based subcellular localization prediction) was employed to predict the subcellular localization of proteins (Table S1). The result indicated that BmGαq, BmPLCβ1, and BmPLCβ4 were situated in the cytoplasm, suggesting they were all predicted to be intracellular proteins. Previous predictions of transmembrane domains revealed that BmDUOX possesses six transmembrane domains, while subcellular localization prediction of Wegoloc indicated that BmDUOX was localized on the plasma membrane of the cell, leading to the presumption that BmDUOX was a plasma membrane protein functioning at the membrane. The result of subcellular localization prediction was supported by the subsequent prediction results of DeepLoc-2.1 and Euk-mPLoc 2.0. The predicted location of BmDUOX was consistent with the functional position of the DmDUOX in the *Drosophila* [18], implying that the BmDUOX of *B. mori* may share similar biological functions with the DmDUOX of *Drosophila*. Hence, it was of great significance to further explore the subcellular localization and biological functions of BmDUOX in *B. mori*.

### 3.3. Expression and Detection of BmDUOX_POD and BmDUOX_OM

*BmDUOX_POD* and *BmDUOX_OM* were cloned via PCR using the specific primers mentioned above and inserted into pColdI vector. The resulting recombinant plasmids were verified by PCR, restriction enzyme digestion, and DNA sequencing. PCR and double digestion products yielded specific bands of approximately 1600 bp and 430 bp (Figure S1), consistent with the molecular weight of the target genes. DNA sequencing further confirmed that the inserted sequences matched the target genes sequences completely. These results indicated that the *BmDUOX_POD*-pColdI-positive plasmid and *BmDUOX_OM*-pColdI-positive plasmid were successfully constructed and could be used for subsequent recombinant protein expression and purification.

Subsequently, the positive recombinant plasmids *BmDUOX_POD*-pColdI and *BmDUOX_OM*-pColdI were transformed into *E. coli Rosetta* (DE3) competent cells for further expression. The results of IPTG-induced expression of BmDUOX_POD and BmDUOX_OM revealed specific protein bands at approximately 62 kDa and 18 kDa, respectively (Figure S2A), consistent with their expected molecular weights. Western blotting was then performed using anti-His-Tag antibody as the primary antibody and HRP-conjugated goat anti-mouse IgG as the secondary antibody. The results showed that both induced protein bands were specifically recognized by the anti-His-Tag antibody, with hybridization signals detected at approximately 62 kDa and 18 kDa, confirming that the correct recombinant proteins were obtained (Figure S2B). The *E. coli* cells were disrupted using ultrasonication and then centrifuged to separate the supernatant and sediment, respectively. The result of SDS-PAGE indicated that the recombinant BmDUOX_POD and BmDUOX_OM were present in the sediment (Figure S2C), suggesting that the recombinant proteins were expressed in inclusion bodies. After collecting a substantial amount of the sediment, the recombinant proteins rBmDUOX_POD and rBmDUOX_OM were purified using Ni-NTA affinity chromatography, yielding the target proteins with expected molecular masses of approximately 62 kDa and 18 kDa (Figure S2D), confirming that the both recombinant proteins were successfully purified.

**Figure 2:**
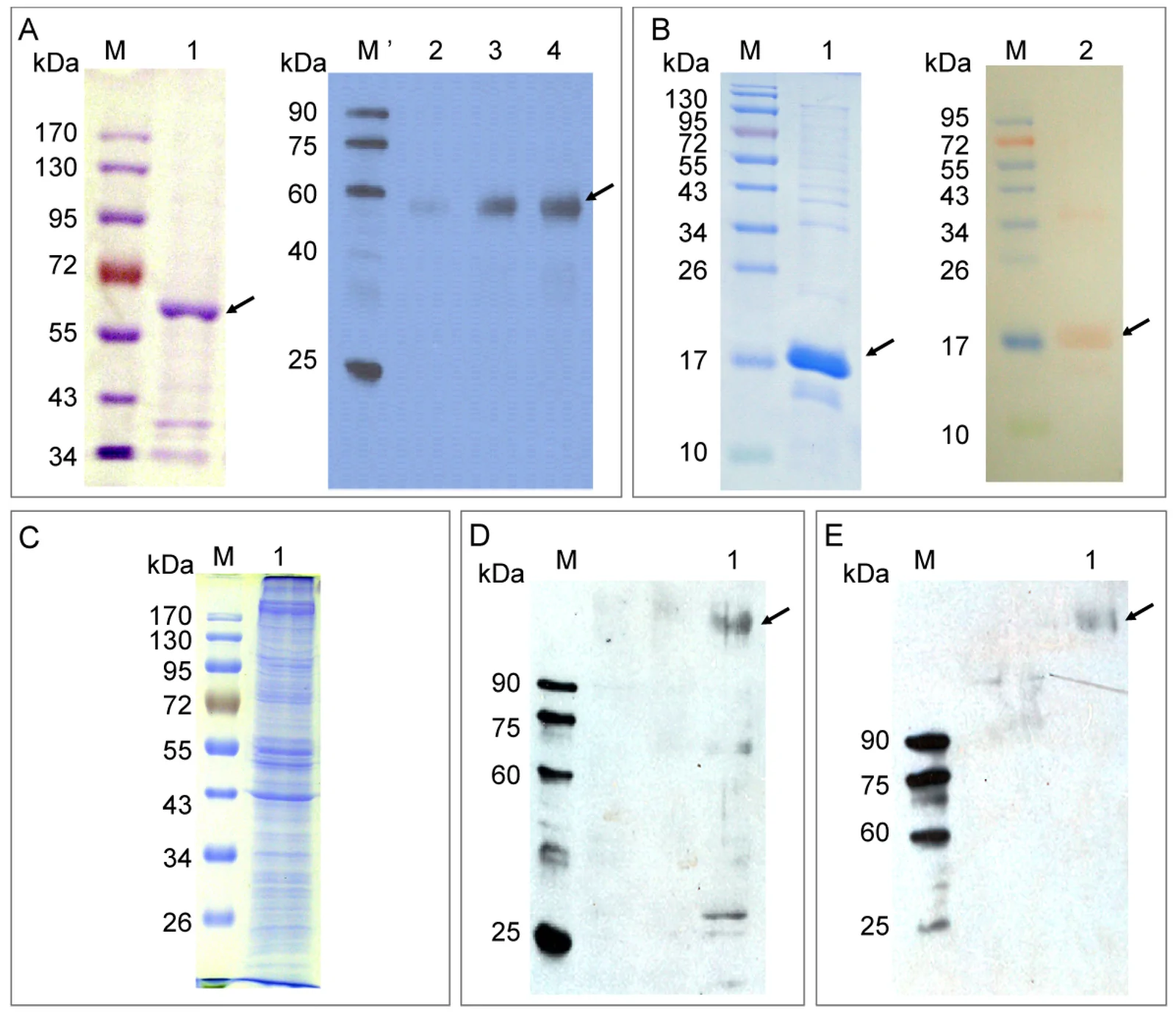
Immunoblotting analysis of anti-BmDUOX_POD and anti-BmDUOX_OM polyclonal antibodies. (**A**) Anti-BmDUOX_POD serum. M: Prestained protein marker; 1: SDS-PAGE analysis of purified rBmDUOX_POD; 2–4: Immunoblotting analysis of anti-BmDUOX_POD serum (under different dilution rates). (**B**) Anti-BmDUOX_OM serum. M: Prestained protein marker; 1: SDS-PAGE analysis of purified rBmDUOX_OM; 2: Immunoblotting analysis of anti-BmDUOX_OM serum. (**C**) Total proteins of silkworm. M: Prestained protein marker; 1: Total proteins of silkworm 3rd of fifth instar larva. (**D**) Anti-BmDUOX_POD serum hybridized to silkworm protein. M: Western Protein Marker; 1: Anti-BmDUOX_POD serum hybridized to total proteins of silkworm 3rd of fifth instar larva. (**E**) Anti-BmDUOX_OM serum hybridized to silkworm protein. M: Western Protein Marker; 1: Anti-BmDUOX_OM serum hybridized to total proteins of silkworm 3rd of fifth instar larva. The black arrow indicated the position of the target protein band.

Polyclonal antibodies against rBmDUOX_POD and rBmDUOX_OM were generated by immunizing mice with purified recombinant proteins and were utilized in the Western blotting analysis. The results showed that specific bands at approximately 62 kDa and 18 kDa (Figure 2A,B) were detected using anti-BmDUOX_POD and anti-BmDUOX_OM antibodies, indicating that these antibodies can specifically recognize the recombinant proteins rBmDUOX_POD and rBmDUOX_OM, respectively. Subsequently, total proteins of silkworm 3rd day of the 5th instar larva were used as antigens and treated with anti-BmDUOX_OM and anti-BmDUOX_POD antibodies. The results indicated that the obtained antibodies were all able to hybridize to a clear target band in the sample lane (Figure 2D,E). The theoretical molecular weight of BmDUOX was approximately 170 kDa, which matches the molecular weight of the hybridized bands. This indicated that the prepared antibodies can specifically recognize BmDUOX in vivo and could be used for subsequent protein localization analysis.

### 3.4. Subcellular Localization Analysis of BmDUOX

#### 3.4.1. BmDUOX Can Be Localized to the Cell Membrane of *B. mori* Midgut Cells

Immunohistochemical analysis was conducted to further explore the subcellular localization of BmDUOX in the midgut tissue of silkworm *B. mori*. Ultra-thin sections of midgut tissue from both healthy *B. mori* and those infected with *N. bombycis* were prepared and stained for observation (Figure S3). The results revealed that the midgut tissue in the sections was relatively intact, facilitating clear observation of midgut cells and the peritrophic matrix, which satisfies the requirements for performing immunohistochemical experiments.

**Figure 3:**
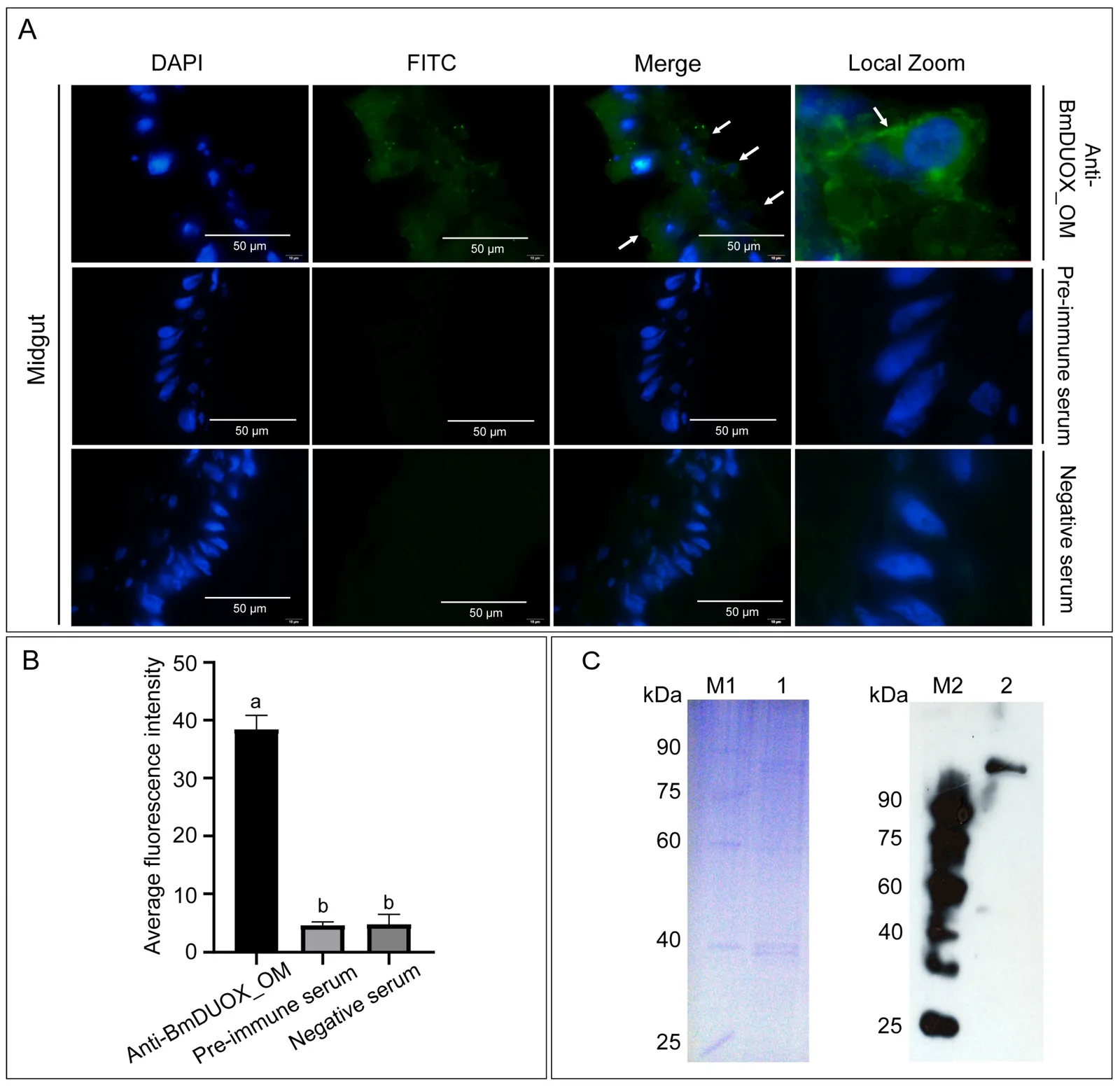
Localization analysis of BmDUOX in silkworm midgut cells. (**A**) Immunohistochemical analysis of BmDUOX in silkworm larva midgut (200×). DAPI was employed to stain the nuclei of silkworm midgut cells (blue fluorescence). Anti-BmDUOX_OM serum was used as the primary antibody in the experimental group. Mouse negative serum was employed as the primary antibody in the negative group. The white arrows indicated the location of the bright green fluorescence, which is the BmDUOX positioning signal. Scale bars = 50 μm. (**B**) Analysis of average fluorescence intensity for the midgut immunofluorescence. Different lowercase letters on the columns indicated significant differences (*p* < 0.05). Statistical significance was determined by Student’s *t*-test. Data are presented as mean ± SEM from three independent replicates. (**C**) Immunoblotting detection of BmDUOX in the membrane proteins of silkworm midgut cells. M1: Western Protein Marker; 1: SDS-PAGE analysis of isolated membrane proteins of midgut cells; M2: Western Protein Marker; 2: Anti-BmDUOX_OM serum hybridized to the membrane proteins of midgut cells.

The immunohistochemical analysis results of the *B. mori* midgut tissue indicated that in the negative control group, no distinct green fluorescence signals were detected in the cellular regions, while the cell nuclei were stained blue (Figure 3A). In contrast, midgut cells treated with anti-BmDUOX_OM serum displayed significant green fluorescence signals in the cellular regions, with stronger signals near the cell edges. The fluorescence density analysis results showed that the anti-BmDUOX_OM serum treated group exhibited higher average fluorescence intensity than the negative serum treated group and pre-immune serum treated group (Figure 3B). The Western blotting result by probing midgut membrane proteins with anti-BmDUOX_OM serum showed a distinct hybridization band in the corresponding lane (Figure 3C). This furthermore indicated that BmDUOX could be expressed in the membrane of the silkworm midgut cells and it was a membrane protein of *B. mori* midgut cells.

#### 3.4.2. BmDUOX Can Be Localized to the Cell Membrane of *B. mori* BmE Cells

Indirect Immunofluorescence Assay (IFA) was utilized to investigate the subcellular localization of BmDUOX in normal cultured BmE cells of *B. mori*. Before proceeding with IFA, the transcription of the *BmDUOX* in BmE cells was assessed. As shown in Figure S4, BmDUOX was consistently transcribed in BmE cells from days 1 to 6 post-passaging, confirming that these cells express endogenous BmDUOX and thus can be used as a valid cellular model for subsequent immunofluorescence-based localization analysis.

**Figure 4:**
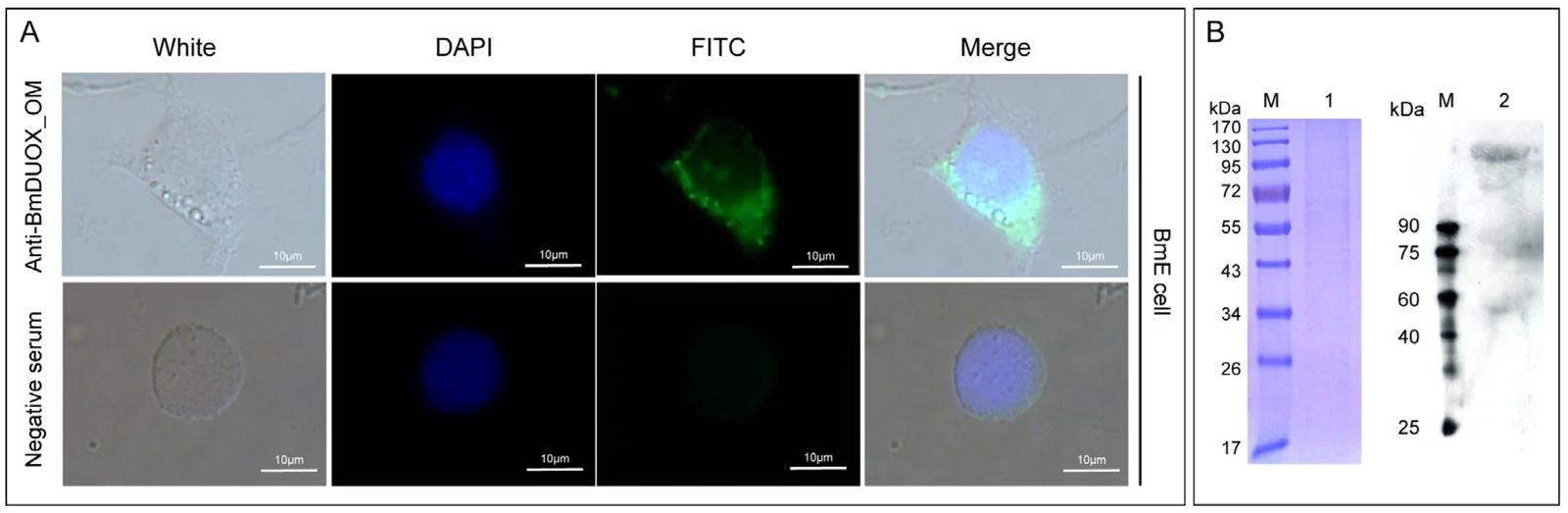
Localization analysis of BmDUOX in BmE cells. (**A**) IFA of BmDUOX in BmE cells. Green fluorescence (FITC) indicated the localization of BmDUOX in BmE cells. DAPI was employed to stain the nuclei of BmE cells (blue fluorescence). Anti-BmDUOX_OM serum was used as the primary antibody in the experimental group. Mouse negative serum was employed as the primary antibody in the negative group. Scale bars = 10 μm. (**B**) Immunoblotting detection of BmDUOX in the membrane proteins of BmE cells. M1: Prestained protein marker; 1: SDS-PAGE analysis of isolated membrane proteins of BmE cells; M2: Western Protein Marker; 2: Anti-BmDUOX_OM serum hybridized to the membrane proteins of BmE cells.

The subcellular localization results of BmDUOX in BmE cells indicated that only the nuclei were stained blue, with no significant green fluorescent spots detected in other areas of the negative control group. In contrast, clear green fluorescence was observed in BmE cells incubated with the anti-BmDUOX_OM serum, displaying relatively strong signals near the cell edges, while no hybridization signals were detected in the nuclear region (Figure 4A). Meanwhile, the Western-blotting results indicated that the BmDUOX protein could be hybridized to the membrane proteins of BmE cells (Figure 4B). Hence, the results confirmed that BmDUOX can locate on the plasma membrane of *B. mori* BmE cells, aligning with the previously reported localization of BmDUOX in *B. mori* BmN cells [36].

### 3.5. The Expression of BmDUOX Gene Was Up-Regulated After N. bombycis Infection

**Figure 5:**
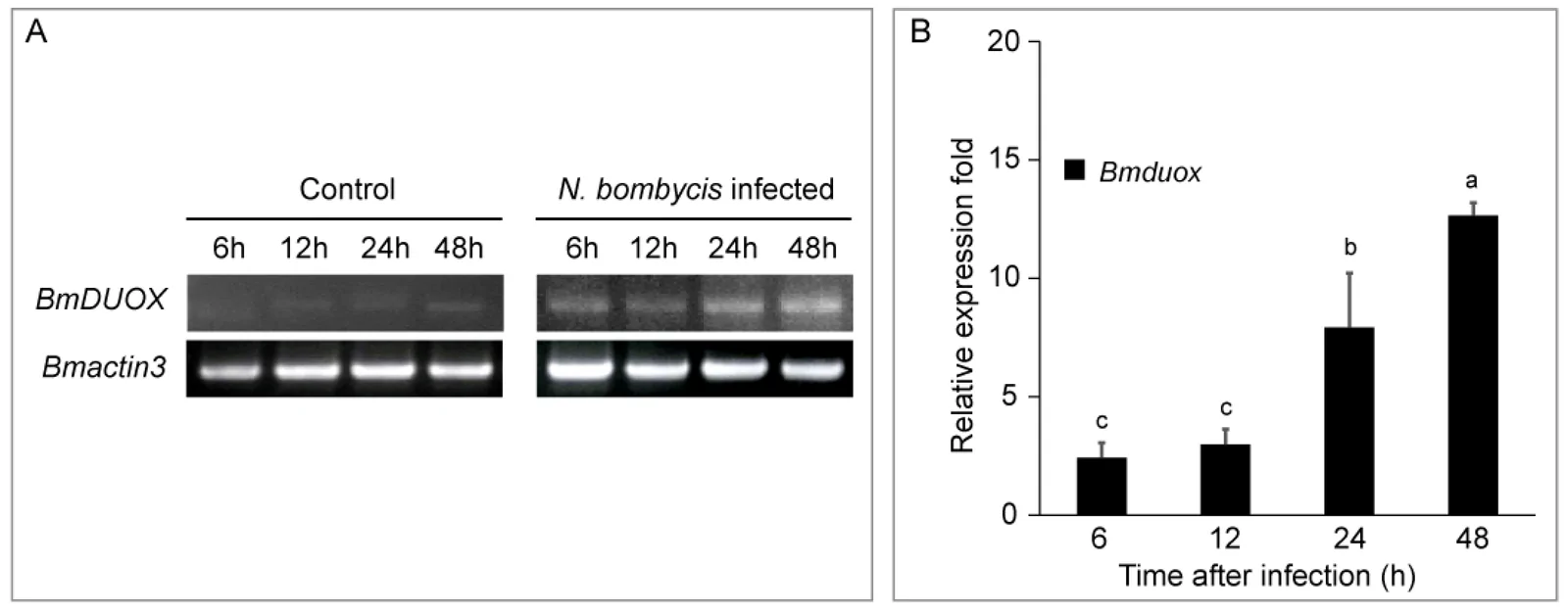
The expression profile of *BmDUOX* after *N. bombycis* infection. (**A**) The results of sqRT-PCR for *BmDUOX* and *Bmactin3*. (**B**) The fold of gene relative expression level of *BmDUOX* in the silkworm midgut after *N. bombycis* infection. Different lowercase letters on the columns indicated significant differences (*p* < 0.05). Statistical significance was determined by Student’s *t*-test. Data are presented as mean ± SEM from three independent replicates, and the different lowercase letters (a, b, c,) above the bars indicate significant differences between groups (*p* < 0.05); bars sharing the same letter are not significantly different.

To compare whether the expression of *BmDUOX* was induced by *N. bombycis*, and to further elucidate the role of *BmDUOX* in the immune response to *N. bombycis*, PCR and sqRT-PCR were employed to analyze the transcription level of *BmDUOX* in midgut samples of *B. mori* infected by *N. bombycis* (Figure 5). The RT-PCR of *BmDUOX* demonstrated that its expression level was significantly up-regulated compared to the control group from 6 to 48 h after *N. bombycis* infection, with a significant positive correlation to the duration of infection. Additionally, the expression levels of other main members of DUOX-ROS pathway, including *BmGαq*, *BmPLCβ1*, *BmPLCβ4*, were also analyzed (Figure S5). The results indicated that following *N. bombycis* infection, all genes except *BmPLCβ1* were amplified to varying degrees in the midgut samples. Compared to the control group that was fed normally, the expression of the *BmGαq* gene was significantly up-regulated at all stages of infection. Its expression level increased initially and continued to rise, peaking at 48 h post-infection. The induction results for the *BmPLCβ4* gene revealed that the effect of *N. bombycis* on *BmPLCβ4* was minimal in the early stages of infection, with the peak upregulation occurring only at 48 h post-infection. These data suggested that *N. bombycis* invasion can induce the activation of the DUOX-ROS pathway and markedly up-regulate *BmDUOX* expression in the host midgut, likely driving the release of ROS from the intestinal epithelial cells of *B. mori*.

### 3.6. The ROS Levels in the BmE Cells Can Be Significantly Up-Regulated by N. bombycis

#### 3.6.1. *N. bombycis* Spores Can Induce ROS Production in the BmE Cells

**Figure 6:**
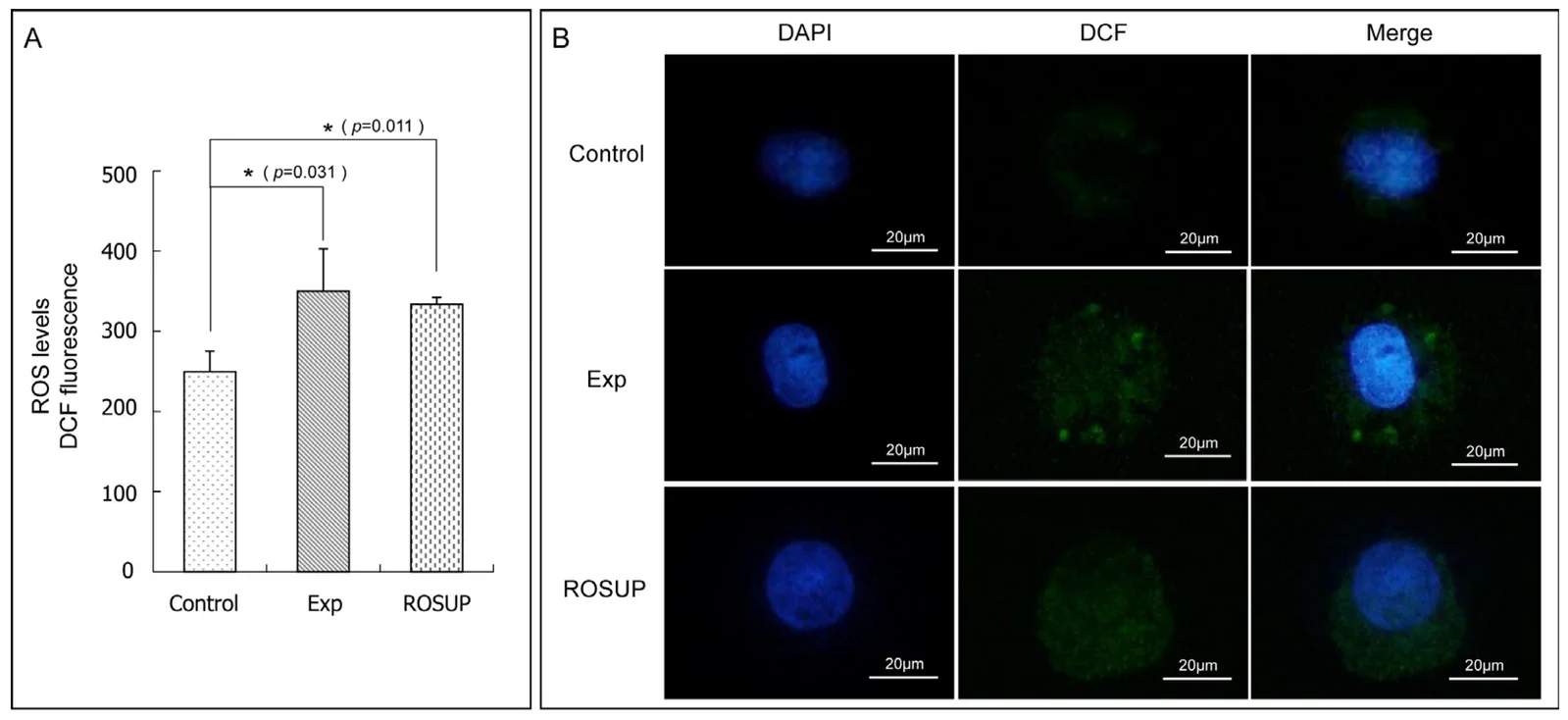
The analysis of ROS production of BmE cells challenged by *N. bombycis* infection. (**A**) Analysis of the production of ROS in BmE cells. Control: Negative control group; Exp: *N. bombycis* treated group; ROSUP: Positive control group. (**B**) Detection of the production of ROS in BmE cells. Green fluorescence (DCF group) indicated the ROS produced by BmE cells. DAPI was employed to stain the nuclei of BmE cells (blue fluorescence). Control: Negative control group; Exp: *N. bombycis* treated group; ROSUP: Positive control group. Statistical significance was determined by Student’s *t*-test. Data are presented as mean ± SEM from three independent replicates, and the asterisks indicate significant differences between different groups (* *p* < 0.05).

The experiment of *N. bombycis* induction was conducted to investigate whether the BmE cells would respond to the spore infection. The results of the induction experiment (Figure 6A) showed that after 24 h induction by *N. bombycis*, the ROS level of the *N. bombycis* infection group was significantly higher than that in the negative control group. Simultaneously, the ROS level in the positive control group were also significantly elevated compared to the negative control group. These findings indicate that *N. bombycis* infection can induce the BmE cells to produce ROS in response to the infection. Additionally, a small number of cells from each experimental group were simultaneously taken and observed under a fluorescence microscope after DAPI staining. The results (Figure 6B) revealed that the green fluorescence intensity in the BmE cells infected by *N. bombycis* significantly increased, suggesting that the ROS level in these cells rose considerably following *N. bombycis* infection. Based on these results, it is speculated that *N. bombycis* infection may activate the DUOX-ROS signaling pathway in BmE cells, prompting them to produce a substantial amount of ROS to combat the invasion of *N. bombycis*.

#### 3.6.2. NbSME Can Induce ROS Production in the BmE Cells

**Figure 7:**
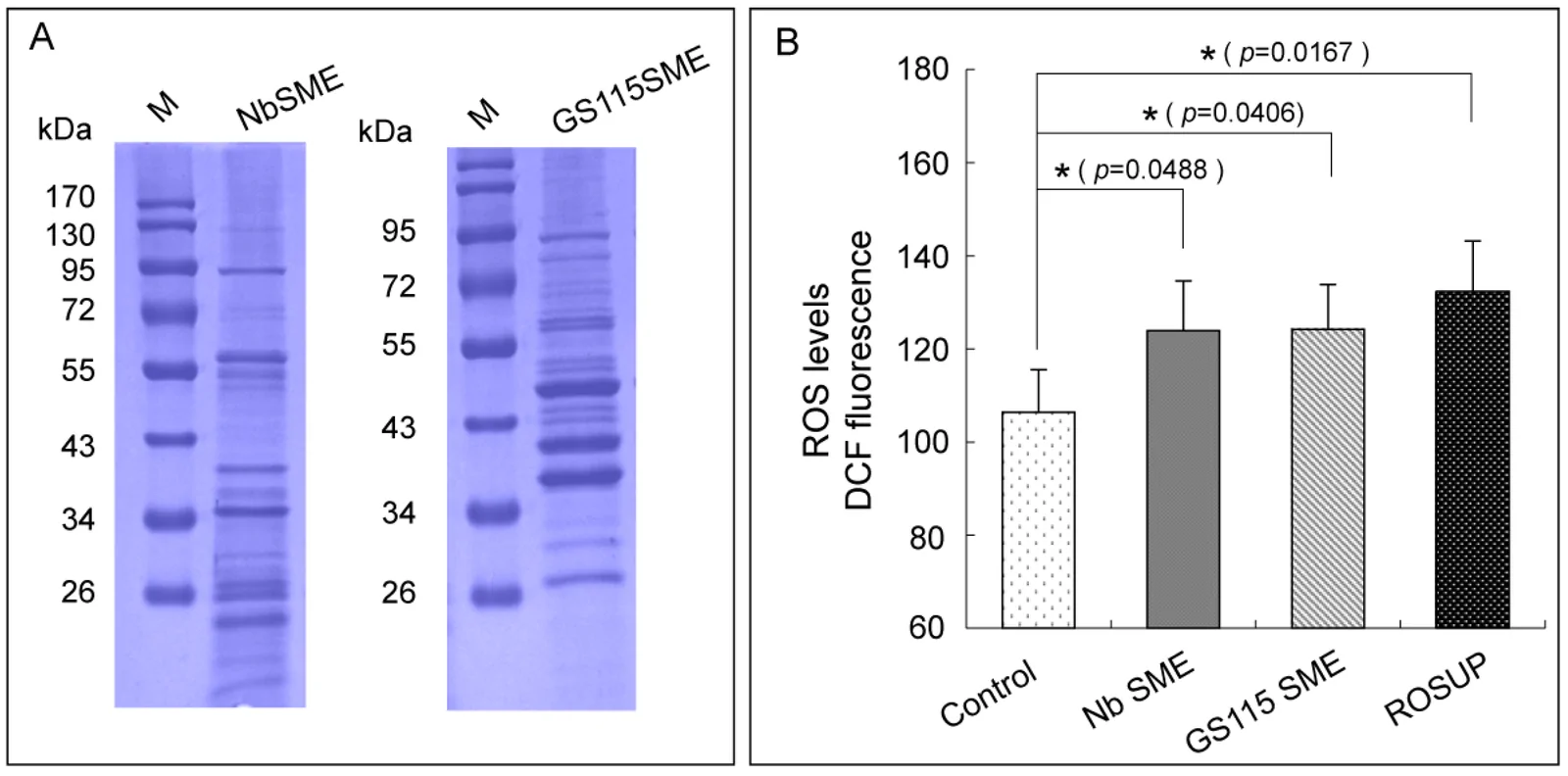
NbSME induce BmE cells. (**A**) Analysis of SMEs using SDS-PAGE. M: Prestained protein marker; NbSME: Soluble Microbial Extract of *N. bombycis*; GS115SME: Soluble Microbial Extract of *Pichia pastoris* (GS115) (**B**) Analysis of the production of ROS in BmE cells. Control: Negative control group; NbSME: Treated by NbSME; GS115SME: Treated by GS115SME; ROSUP: Positive control group. Statistical significance was determined by Student’s *t*-test. Data are presented as mean ± SEM from three independent replicates. Asterisks indicate statistically significant differences (* *p* < 0.05).

The former results indicated that *N. bombycis* mature spores could significantly stimulate BmE cells to release ROS for immunity. To further investigate whether *N. bombycis* components can induce ROS production in BmE cells, this study utilized NbSME to stimulate the BmE cells and measured the ROS levels. Previous studies have confirmed that GS115SME can induce the release of Ca^2+^ from *Drosophila* S2 cells, subsequently regulating DUOX to control ROS production [20]. Therefore, GS115SME was employed as an internal reference in this study. NbSME and GS115SME were extracted, with the soluble microbial extract protein concentration determined at 1.0 mg/mL using the Bradford method, and the protein richness of the sample assessed by SDS-PAGE. The SDS-PAGE results (Figure 7A) show that the protein bands in the SME are clear and abundant, suitable for subsequent experiments. The SME induction experiment results (Figure 7B) demonstrated that after stimulation with NbSME and GS115SME, the ROS content in BmE cells significantly increased compared to the control group, with the up-regulation level of ROS being roughly equivalent. Additionally, the ROS level in the positive control group was also significantly up-regulated compared to the control group. These findings indicate that both NbSME and GS115SME can activate the DUOX-ROS signaling pathway in *B. mori* cells, inducing substantial ROS production. This suggests that the molecular mechanism of immunity to *N. bombycis* infection via the DUOX-ROS signaling pathway in *B. mori* may be similar to that in *Drosophila*.

### 3.7. BmDUOX Can Mediate the Resistance of BmE Cells to N. bombycis Infection

**Figure 8:**
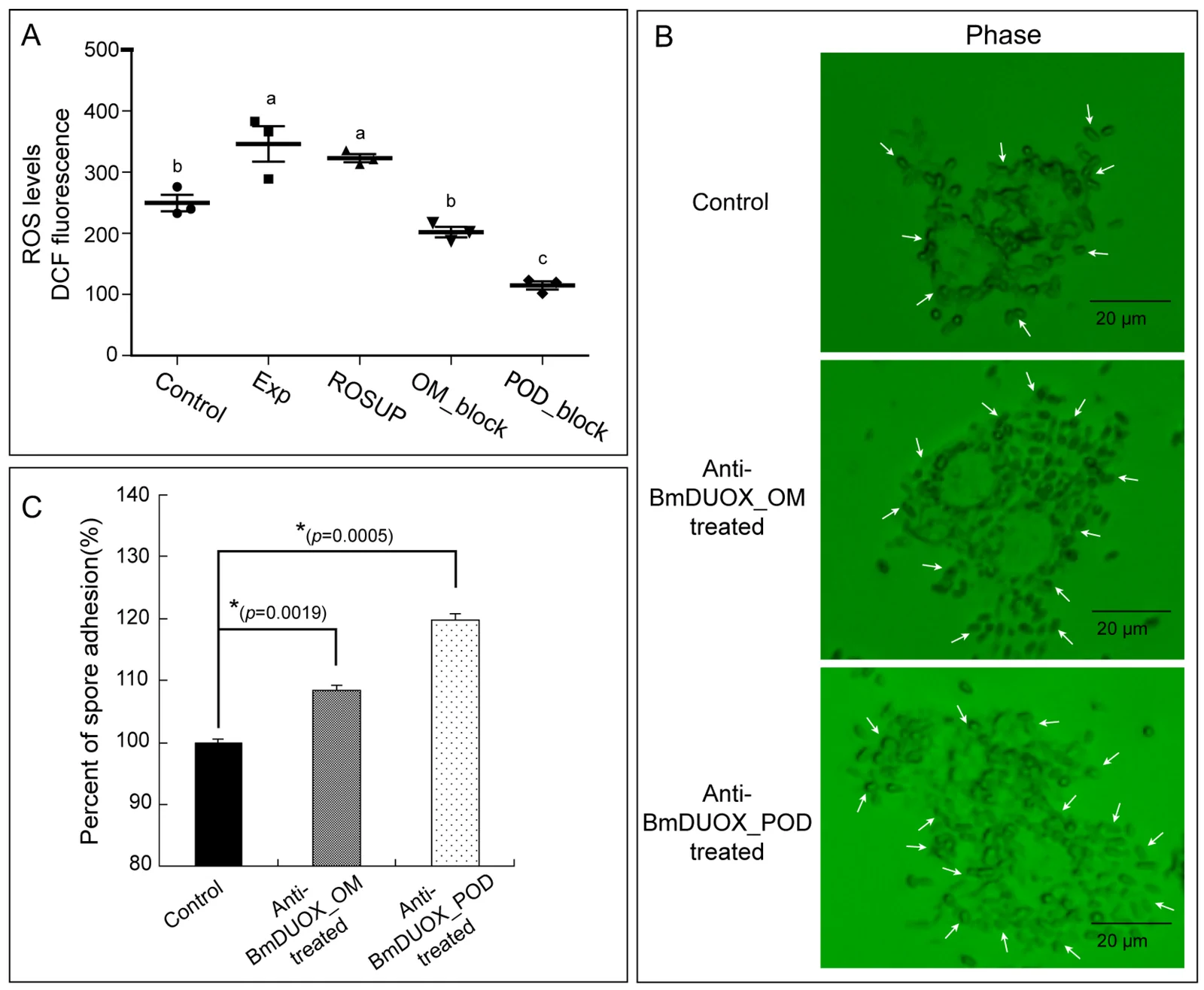
Effect of BmDUOX-specific antibody on ROS production level and *N. bombycis* adhesion rate to BmE cells at 2 hpi. (**A**) Analysis of the ROS production level in BmE cells. Control: Negative control group; Exp: *N. bombycis* treated group; ROSUP: Positive control group; OM_block: BmDUOX_OM was blocked by anti-BmDUOX_OM serum then treated with *N. bombycis*; POD_block: BmDUOX_POD was blocked by anti-BmDUOX_POD serum then treated with *N. bombycis*. Statistical significance among the five groups was determined by one-way ANOVA followed by Tukey’s post-hoc test. Data are presented as mean ± SEM from three independent replicates. Different lowercase letters above bars indicate significant differences among multiple groups (*p* < 0.05). (**B**) Observation of *N. bombycis* spores adhering to the BmE cells. Control: BmE cells treated with mouse negative serum; Anti-BmDUOX_OM treated: BmE cells treated with Anti-BmDUOX_OM serum; Anti-BmDUOX_POD treated: BmE cells treated with Anti-BmDUOX_POD serum. The white arrows indicate the spores of *N. bombycis*. (**C**) Statistic analysis of adhesion rate of *N. bombycis* spores to the BmE cells. Control: BmE cells treated with mouse negative serum; Anti-BmDUOX_OM treated: BmE cells treated with Anti-BmDUOX_OM serum; Anti-BmDUOX_POD treated: BmE cells treated with Anti-BmDUOX_POD serum. Statistical significance between groups was determined by Student’s *t*-test. Data are presented as mean ± SEM from three independent replicates. Asterisks indicate statistically significant differences (* *p* < 0.05).

Antibody blocking experiments were conducted to test the immune function of BmDUOX in the infection process of *N. bombycis*. As shown in Figure 8A, the ROS level of the *N. bombycis* infection group was significantly higher than that in the negative control group and did not differ significantly from that in the positive control group, consistent with previous results. However, the OM_block group and the POD_block group in which different domains of BmDUOX were blocked by anti-BmDUOX_OM serum or anti-BmDUOX_POD serum, showed a significant decrease in the ROS level compared with the *N. bombycis* infection group, indicating that BmDUOX may act as a crucial component in the DUOX-ROS signaling pathway of *B. mori*.

After adding *N. bombycis* spores to the cell culture plate for 2 h, a greater number of *N. bombycis* spores adhered around the experimental group cells treated with anti-BmDUOX_OM serum or anti-BmDUOX_POD serum, demonstrating a significant difference compared to the negative control group cells treated with mouse negative serum (Figure 8B,C). Meanwhile, in comparison to the anti-BmDUOX_OM serum treatment group, the increase in *N. bombycis* adhesion rate in the anti-BmDUOX_POD serum treatment group was more pronounced. Results from the antibody blocking experiments indicate that the inactivation of BmDUOX facilitates *N. bombycis* spore adhesion to host cells, suggesting that BmDUOX plays a crucial role in the initial stage of host cell resistance to *N. bombycis* infection. Additionally, the peroxidase domain of BmDUOX may be an essential participant in this process.

**Figure 9:**
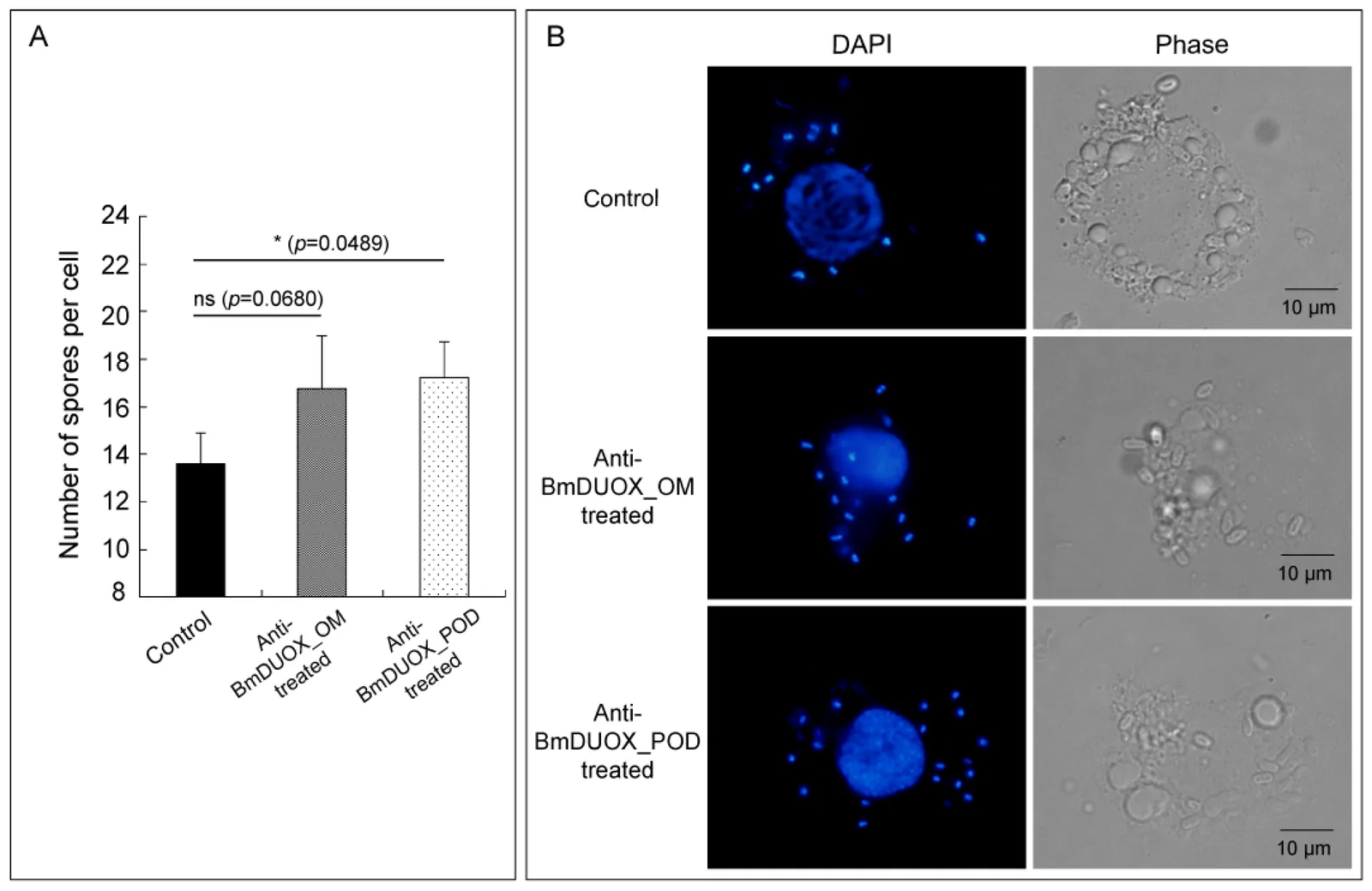
Effect of BmDUOX-specific antibody on *N. bombycis* invasion to BmE cells at 2 days post-infection (dpi). (**A**) The number of *N. bombycis* spores that have invaded in per cell. Control: BmE cells treated with mouse negative serum; Anti-BmDUOX_OM treated: BmE cells treated with Anti-BmDUOX_OM serum; Anti-BmDUOX_POD treated: BmE cells treated with Anti-BmDUOX_POD serum. (**B**) Observation of *N. bombycis* spores invading to the BmE cells. DAPI was employed to stain the nuclei of BmE cells (blue fluorescence). Statistical significance was determined by Student’s *t*-test. Data are presented as mean ± SEM from three independent replicates. Asterisks indicate statistically significant differences (* *p* < 0.05); ns, not significant.

Consequently, on the second day post-infection, we utilized DAPI to stain the nuclei of the BmE cells and counted the number of spores that had entered the host cells. As shown in Figure 9, the treatment of the BmE cells with anti-BmDUOX antibody resulted in significantly more spores invading host cells compared to the control group. Notably, after treatment with anti-BmDUOX_POD serum, the number of *N. bombycis* spores entering the host cells was significantly higher than that in the control group. This indicates that when the BmDUOX on the cell surface is blocked, it not only facilitates the adhesion of spores to the host cells but also promotes the invasion of spores into those cells. Based on the results above, we speculate that BmDUOX may play a significant role in the primary reactions of *B. mori* resistance to *N. bombycis* infection, with the peroxidase domain of BmDUOX likely serving as the key functional domain.

## 4. Discussion

The intestinal epithelium is the primary tissue in insects that most frequently interacts with microorganisms [4]. It plays a crucial biological role in maintaining the stability of intestinal symbiotic microbial communities and resisting the invasion of foreign pathogenic microorganisms. For insects, it is a delicate task for the host to combat infectious microbes through immune responses while preserving the homeostasis of intestinal symbiotic microorganisms [18,21]. This requires that the insect gut mount immune responses against pathogens while tolerating the normal gut microbiota and dietary microorganisms. Previous studies on the immune response mechanisms in *Drosophila* have demonstrated that the fly immune response primarily relies on two types of antimicrobial molecules—AMPs and ROS—which work synergistically to limit the growth and proliferation of invading pathogenic microbes [7,8]. Notably, the ROS production system mediated by the DUOX protein is fundamental to the gut’s antibacterial response, as it not only maintains the homeostasis of the *Drosophila*’s intestinal symbiotic microbial community but also plays a vital role in clearing invading pathogenic microbes from the gut [13,20]. Existing research has shown that, in addition to *Drosophila*, DUOX proteins in various organisms play a crucial role in immune defense and maintaining intestinal homeostasis. For instance, the DUOX protein in zebrafish functions similarly to *Drosophila* DUOX in intestinal immune defense response [38]; human DUOX is expressed in the thyroid and respiratory epithelium, where it is involved in processes such as thyroid hormone synthesis and respiratory epithelial immune responses [39], suggesting that DUOX may play conserved roles across different species. Consequently, the BmDUOX protein may also play a vital role in eliminating pathogenic microorganisms that invade the intestine.

In this study, we found that *B. mori* also utilizes the DUOX-dependent immune response as a defense in the gut as previously characterized in *Drosophila* [13,18]. This research is the first to systematically analyze the molecular components of the DUOX-ROS pathway and its role in resisting *N. bombycis* infection in *B. mori*. Through homologous sequence retrieval, we identified four key members of the DUOX-ROS pathway: *BmGαq*, *BmPLCβ1*, *BmPLCβ4*, and *BmDUOX*, in the *B. mori* genome database. We analyzed and predicted the basic properties of these genes using bioinformatics methods. BmGαq, BmPLCβ1, and BmPLCβ4 were predicted to be cytoplasmic proteins, while BmDUOX was predicted to be localized to the plasma membrane. Domain analysis revealed that the domains of BmGαq, BmPLCβ1, BmPLCβ4, and BmDUOX proteins are highly similar to those of their *Drosophila* homologs, suggesting evolutionary conservation of the DUOX-ROS pathway in insects [40]. Notably, subcellular localization results indicated that BmDUOX is localized on the plasma membrane of the *B. mori* midgut tissue and BmE cells, consistent with the localization site of *Drosophila* DmDUOX [18], suggesting they may share conserved biological functions. Meanwhile, multiple sequence alignment analysis of the peroxidase domain (BmDUOX_POD) in BmDUOX revealed that the BmDUOX_POD sequence shows high conservation in both substrate-binding and heme-binding sites within the POD domain across various species. Studies have demonstrated that the peroxidase domains of human hDUOX2 and *Caenorhabditis elegans* CeDUOX1 can convert superoxide into H_2_O_2_ [41,42]. This indicates that BmDUOX_POD may also share a similar function, specifically participating in the conversion of superoxide to H_2_O_2_ in *B. mori* cells. Consequently, in-depth analysis of the clearance and killing effects of H_2_O_2_ on pathogenic microorganisms, both in vivo and in vitro, has emerged as a crucial research area for further understanding the intestinal immune mechanism of *B. mori*.

When *Drosophila* are infected by pathogens, the midgut swiftly releases ROS to combat the invaders, a process typically regulated by the DUOX protein. The activation of the DUOX protein, which produces substantial amounts of ROS to eliminate pathogens during invasion, is mediated by the Gαq-PLCβ-Ca^2+^ signaling pathway [20]. This regulatory mechanism often relies on immune pattern recognition receptors that identify components of the microbial cell wall [43], while the activation of the Gαq-PLCβ-Ca^2+^ pathway relies on ligands secreted by the pathogens, specifically non-PG (uridine). As a legally designated quarantine target in sericulture, *N. bombycis* can lead to significant economic losses in *B. mori* production due to the microsporidian diseases it induces [44]. Current research on domestic *N. bombycis* primarily focuses on structural proteins [45], infection-related proteins [46,47], and host responses triggered by the pathogens [29,48]. However, studies examining the immune mechanisms of the domestic *B. mori* in response to microsporidia from the host’s perspective are extremely rare. Recent studies have begun to shed light on this aspect. For instance, BmTSPO was found to be upregulated upon *N. bombycis* infection and to activate innate immune signaling pathways (IMD and JAK-STAT) to inhibit pathogen proliferation [49]. Additionally, *N. bombycis* has been shown to actively manipulate host immunity by secreting NbSPN14, a serine protease inhibitor that suppresses host cell apoptosis via direct inhibition of Caspase BmICE [50]. Therefore, in this study, we conducted pathogen-induced experiments to assess the expression of *B. mori* DUOX-ROS pathway members under *N. bombycis* induction. The results indicated that *N. bombycis* infection significantly upregulated the expression of *BmGαq* and *BmDUOX*, which raises the possibility that *B. mori* may activate the DUOX-ROS pathway via the Gαq-PLCβ-Ca^2+^ pathway, thereby mediating ROS production to combat *N. bombycis* infection. This mechanism resembles the “A microbe-derived ligand activates G protein-coupled receptor (GPCR)-Ca^2+^ signal” model in *Drosophila* [20], but whether *B. mori* relies on similar ligands requires further verification. Concurrently, we confirmed that both *N. bombycis* spores and their soluble extracts (NbSME) significantly elevated ROS levels in BmE cells. These findings suggest that following *N. bombycis* infection, the host recognizes *N. bombycis* and relies on a specific substance in NbSME to activate the DUOX-ROS signaling pathway, synthesize and release ROS, thereby establishing the initial line of defense against *N. bombycis* invasion in the intestinal epithelium. It should be noted that DCFH-DA is sensitive to diverse cellular oxidative reactions, and the fluorescence signals cannot be exclusively attributed to H_2_O_2_ derived from BmDUOX peroxidase activity. Additional specific probes such as Amplex-Red will be adopted in future investigations to more precisely characterize BmDUOX-dependent H_2_O_2_ generation.

Currently, the most widely accepted microsporidia infection hypothesis suggests that microsporidia invasion of host cells begins with the adhesion of the spore to the cell [51,52], and the spore wall proteins may play a crucial role during the interaction of microsporidia and host cells [53]. Existing studies have shown that *N. bombycis* exhibits phenomena associated with adhesion to host cells during the infection process, with its spore wall proteins playing a role in the adhesion of *N. bombycis* spores to host cells, thereby initiating subsequent infection processes [46,54]. Recent studies have identified additional spore wall proteins involved in this process, including NbSWP16, a 44 kDa exospore protein with proline-rich tandem repeats that participates in spore adherence to host cells [55], and NbSWP26, which has been shown to bind with the turtle-like protein (BmTLP) of *B. mori*, thereby facilitating spore invasion into silkworm cells [31]. After adhering to the surface of the host cell, microsporidia will rapidly extrude the polar tube and inject the infectious sporoplasm into the host cell when exposed to appropriate environmental stimulation [51,52,56,57]. The polar tube, a highly specialized invasion apparatus of microsporidia, is primarily composed of polar tube proteins (PTPs). The biological functions of the polar tube largely rely on these proteins [52,58,59,60,61,62]. PTPs are essential during the invasion of microsporidia into the host organism. For example, PTP4 has been shown to interact with the host cell TfR1, and NbPTP6 has also been demonstrated to adhere to the host cell surface, indicating a potential role in facilitating the invasion process of microsporidia [52,61].

Antibody blocking assay demonstrated that inhibiting BmDUOX with anti-BmDUOX_OM serum or anti-BmDUOX_POD serum significantly improved the efficiency of *N. bombycis* spore adhesion to and invasion of host cells. Based on these findings and the microsporidia infection hypothesis, we propose a putative mechanism by which the *B. mori* DUOX-ROS signaling pathway defends against *N. bombycis* infection: *N. bombycis* infection may activate the *B. mori* Gαq-PLCβ-Ca^2+^ signaling pathway, which in turn could trigger the DUOX-expression pathway, upregulating the expression of key proteins such as BmDUOX and prompting *B. mori* cells to produce large amounts of ROS. Our observation that blocking BmDUOX promoted spore adhesion and invasion supports the hypothesis that ROS may compromise spore functional integrity. Given that spore wall proteins and polar tube proteins play critical roles in spore adhesion and host cell invasion [46,52,54,61], it is plausible that ROS affects these spore surface components to impair their function. However, the causal relationship between BmDUOX-derived ROS and inhibition of *N. bombycis* spore adhesion and invasion was supported only by antibody-blocking assays in the present study. Complementary genetic evidence, such as RNAi or CRISPR-mediated gene knockout together with rescue experiments, is still needed for more rigorous validation, which will be prioritized in our future investigations. Additionally, it is worth mentioning that more work is needed to explore how host cells balance the effect of ROS generation to resist pathogens and the damage to their own cells, whether the concentration of hydrogen peroxide in cells in vivo can effectively inhibit spore germination, and the specific mechanism of ROS inhibiting spore germination and infection, which has also become a main focus of our follow-up research.

## 5. Conclusions

In this study, we systematically identified and characterized four core components of the DUOX-ROS pathway in *Bombyx mori*—*BmGαq*, *BmPLCβ1*, *BmPLCβ4*, and *BmDUOX*—and demonstrated their possible involvement in the intestinal immune response against *Nosema bombycis* infection. Our results showed that *N. bombycis* infection significantly upregulated *BmDUOX* expression and induced ROS production in the silkworm midgut. Immunofluorescence and antibody-blocking assays confirmed that BmDUOX localized to the plasma membrane and played a critical role in restricting spore adhesion and invasion. These findings provided the first systematic evidence of DUOX-ROS-mediated anti-microsporidian immunity in *B. mori*, filling a gap in the understanding of intestinal immune mechanisms in economically important insects. Furthermore, this study may provide a theoretical foundation for future exploration of ROS-based disease prevention strategies in sericulture.

## Figures and Tables

**Table 1:** Characterization of the members involved in DUOX-ROS pathway in *B. mori*.

Gene Name	SilkDB ID	Chromosome No.	Number of Exons	Signal Peptide /Transmembrane Domain	Number of Amino Acids	Molecular Weight/kDa	pI
*BmGαq*	BMSK0005025	Chr.9	9	No/No	353	41.49	5.15
*BmPLCβ1*	BMSK0007255	Chr.12	18	No/No	952	107.86	6.72
*BmPLCβ4*	BMSK0010460	Chr.18	16	No/No	919	106.10	6.76
*BmDUOX*	BMSK0004484	Chr.8	26	No/Yes(6)	1428	164.14	8.88

## Data Availability

Data is contained within the article or Supplementary Materials. Further inquiries can be directed to the corresponding author.

## Supplementary Materials

The following supporting information can be downloaded at https://www.mdpi.com/article/10.3390/biology15171544/s1: Figure S1. Verification of recombinant plasmid; Figure S2. Expression and purification of recombinant proteins rBmDUOX_POD and rBmDUOX_OM; Figure S3. Tissue biopsies of silkworm midgut (200×); Figure S4. Transcriptional profile of *BmDUOX* in BmE cell; Figure S5. The expression profile of DUOX-ROS pathway-related genes after *N. bombycis* infection in silkworm midgut; Table S1. Prediction of the subcellular location of main silkworm DUOX-ROS pathway members using Wegoloc tool; Table S2. Full Western blot Figures of the manuscript.

## Abbreviations

The following abbreviations are used in this manuscript:

ROS	Reactive Oxygen Species
DUOX	Dual Oxidase
PLCβ1	Phospholipase C Beta 1
PLCβ4	Phospholipase C Beta 4
Gαq	G-protein Alpha q Subunit
POD	Peroxidase Domain
OM	Outer Membrane Region
PG	Peptidoglycan
PGRPs	Peptidoglycan Recognition Proteins
SME	Soluble Microbial Extract
IFA	Indirect Immunofluorescence Assay
DAPI	4′,6-Diamidino-2-Phenylindole
FITC	Fluorescein Isothiocyanate
DPP	Dapsone
DCFH-DA	2′,7′-Dichlorodihydrofluorescein diacetate
