# Supplementary material for: DUOX-Generated Intestinal ROS Restrict Spore Adhesion and Invasion of Nosema bombycis in Silkworm Bombyx mori
Source: Biology (Basel). 2026 Sep 4;15(17):1544. doi: 10.3390/biology15171544 (PMC13565750; doi:10.3390/biology15171544)

Supplementary material

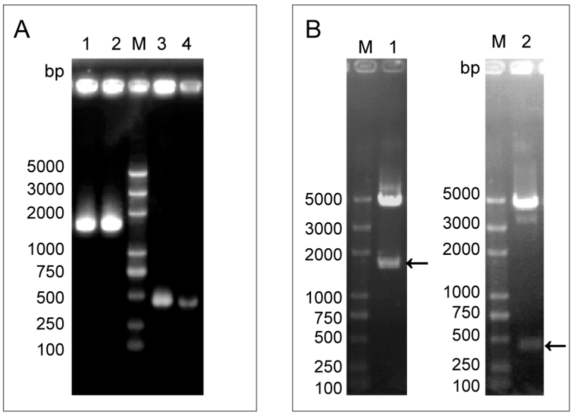

Figure S1. Verification of recombinant plasmid. (**A**) PCR test of recombinant plasmid. M: Trans2K DNA standard; 1,2: Recombinant plasmid of *BmDUOX_POD*-pColdI; 3,4: Recombinant plasmid of *BmDUOX_OM*-pColdI. (**B**) Double Digestion of recombinant plasmid. M: Trans2K DNA standard; 1: Recombinant plasmid of *BmDUOX_POD*-pColdI; 2: Recombinant plasmid of *BmDUOX_OM*-pColdI. The black arrow indicated the target gene fragment.

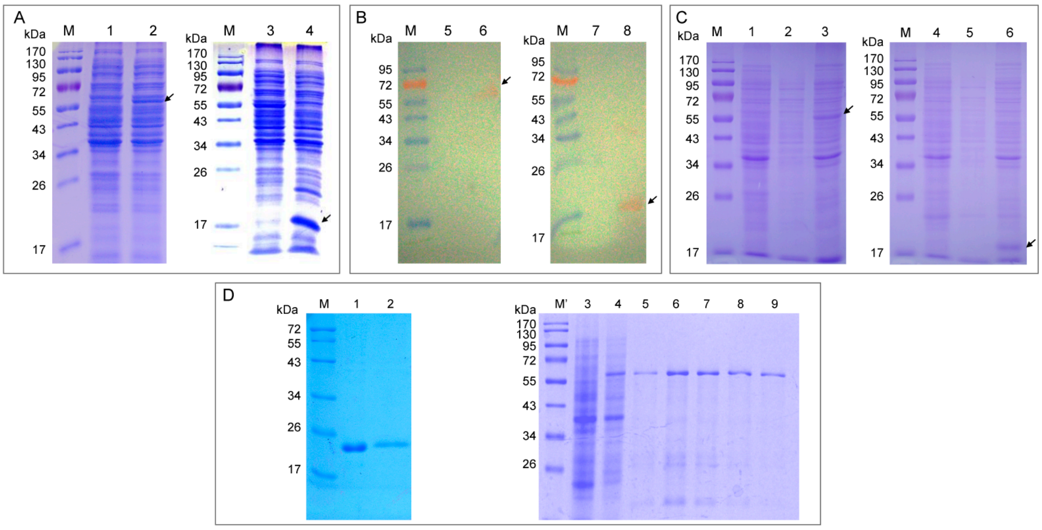

Figure S2. Expression and purificationof recombinant protein rBmDUOX_POD and rBmDUOX_OM. (**A**) Induced Expression analysis. M: Prestained protein marker; 1: Lysate of *BmDUOX_POD*-pColdI recombinant without IPTG; 2: Lysate of *BmDUOX_POD*-pColdI recombinant induced by IPTG; 3: Lysate of *BmDUOX_OM*-pColdI recombinant without IPTG; 4: Lysate of *BmDUOX_OM*-pColdI recombinant induced by IPTG. (**B**) Western Blotting analysis. M: Prestained protein marker; 5: Lysate of *BmDUOX_POD*-pColdI recombinant without IPTG; 6: Lysate of *BmDUOX_POD*-pColdI recombinant induced by IPTG; 7: Lysate of *BmDUOX_OM*-pColdI recombinant without IPTG; 8: Lysate of *BmDUOX_OM*-pColdI recombinant induced by IPTG. (**C**) Expression form analysis. M: Prestained protein marker; 1: Lysate of *BmDUOX_POD*-pColdI recombinant without IPTG; 2: The supernatant of *BmDUOX_POD*-pColdI recombinant after ultrasonication; 3: The sediment of *BmDUOX_POD*-pColdI recombinant after ultrasonication; 4: Lysate of *BmDUOX_OM*-pColdI recombinant without IPTG; 5: The supernatant of *BmDUOX_OM*-pColdI recombinant after ultrasonication; 6: The sediment of *BmDUOX_OM*-pColdI recombinant after ultrasonication. (**D**) Purification of recombinant protein rBmDUOX_POD and rBmDUOX_OM. M: Low molecular weight protein standard; 1~2: rBmDUOX_OM washed from His-affinity chromatography by Elution buffer (under different dilution rate); M′: Prestained protein marker; 3: Lysate of *BmDUOX_POD*-pColdI recombinant without IPTG; 4: Lysate of *BmDUOX_POD*-pColdI recombinant induced by IPTG; 5~9: rBmDUOX_POD washed from His-affinity chromatography by Elution buffer (under different dilution rate). The black arrow indicated the position of the target protein band.

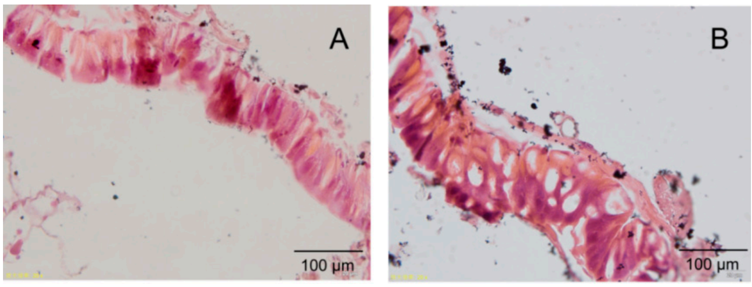

Figure S3. Tissue biopsies of silkworm midgut (200×). (**A**) Midgut of normal feeding silkworm. (**B**) Midgut of *N. bombycis* infected silkworm at 6 days post infection.

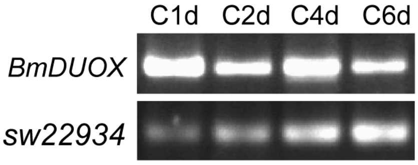

Figure S4. Transcriptional profile of *BmDUOX* in BmE cell. C: Normal cultured BmE cells; d: days after cell passage; *sw22934*: reference gene.

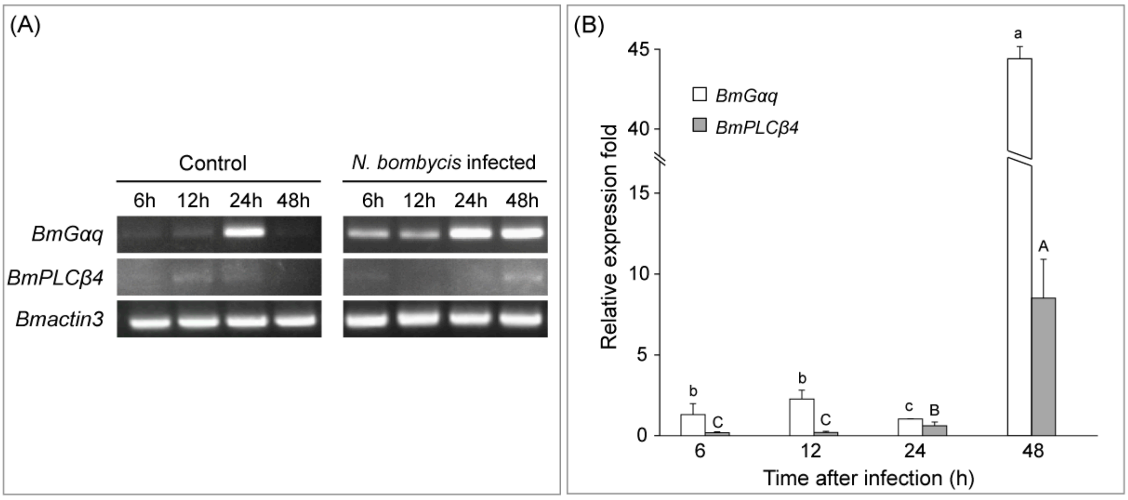

Figure S5. The expression profile of DUOX-ROS pathway related genes after *N. bombycis* infection in silkworm midgut. (**A**) The results of sqRT-PCR for *BmGαq*, *BmPLCβ4* and *Bmactin3*. (**B**) The fold of gene relative expression levels of *BmGαq* and *BmPLCβ4* in the midgut of *B. mori* after *N. bombycis* infection. Statistical significance was determined by Student's t-test. Data are presented as mean ± SE from three independent replicates, and the different lowercase letters (a, b, c,) above the bars indicate significant differences between groups ($p < 0.05$); bars sharing the same letter are not significantly different.

Table S1. Prediction of the subcellular location of main silkworm DUOX-ROS pathway members using Wegoloc tool.

Predicted location	Protein name			
	BmGαq	PLCβ1	PLCβ4	BmDUOX
Plasma membrane	11.1	11.1	11.1	**22.2**
Peroxisome	16.7	11.1	13.9	16.7
Endoplasmic recticulum	8.3	5.6	5.6	16.7
Cytoplasm	**22.2**	**22.2**	**22.2**	13.9
Mitochondrion	5.6	0	0	13.9
Golgi apparatus	0	5.6	2.8	8.3
Lysosom	2.8	16.7	13.9	5.6
Extracelluar region	16.7	19.4	19.4	2.8
Nucleus	16.7	8.3	11.1	0

Note: The data in the table represented the localization probability value of the corresponding protein, and the box marked the maximum probability value.

Table S2. Full Western blot Figures of the manuscript.

Western blot Figure No.	WB figures in the manuscript	Full WB Figures
Fig. 2A	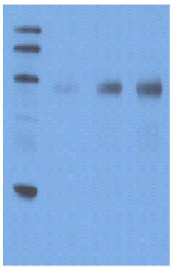	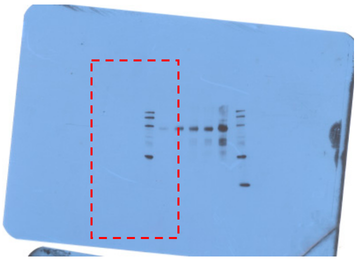
Fig. 2B	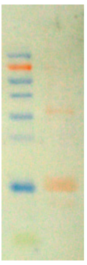	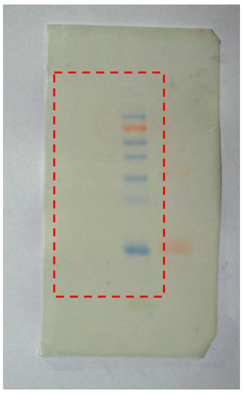
Fig. 2D	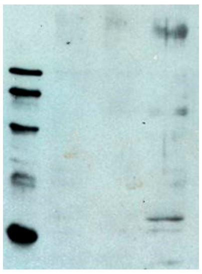	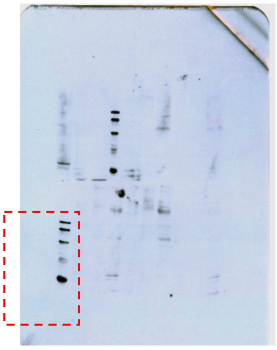
Fig. 2E	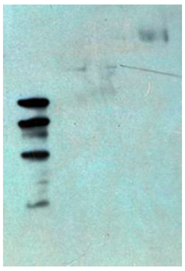	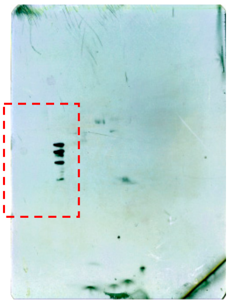

Fig. 3B

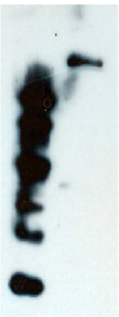

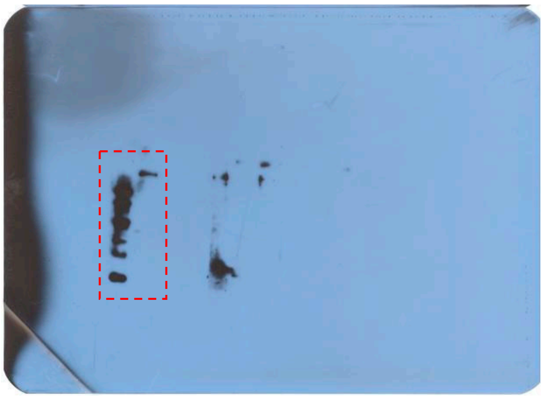

Fig. 4B

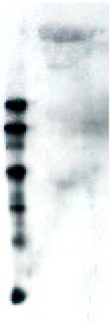

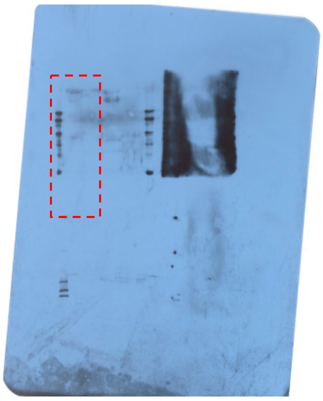

Fig.S2B

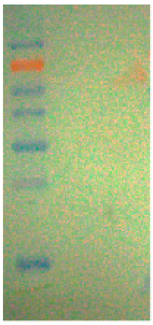

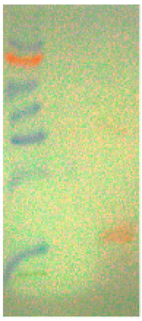

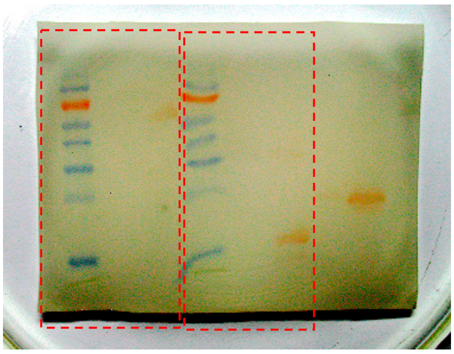

Supplement: Supplementary file 1 [file biology-15-01544-s001.zip › biology-4508802-supplementary.pdf]
